# Chk1 inhibition significantly potentiates activity of nucleoside analogs in TP53-mutated B-lymphoid cells

**DOI:** 10.18632/oncotarget.11388

**Published:** 2016-08-19

**Authors:** Jana Zemanova, Ondrej Hylse, Jana Collakova, Pavel Vesely, Alexandra Oltova, Marek Borsky, Kristina Zaprazna, Marie Kasparkova, Pavlina Janovska, Jan Verner, Jiri Kohoutek, Marta Dzimkova, Vitezslav Bryja, Zuzana Jaskova, Yvona Brychtova, Kamil Paruch, Martin Trbusek

**Affiliations:** ^1^ Department of Internal Medicine – Hematology and Oncology, University Hospital Brno and Faculty of Medicine, Masaryk University, Brno, Czech Republic; ^2^ Center of Biomolecular and Cellular Engineering, International Clinical Research Center, St. Anne's University Hospital, Brno, Czech Republic; ^3^ Department of Chemistry, CZ Openscreen, Faculty of Science, Masaryk University, Brno, Czech Republic; ^4^ Institute of Physical Engineering, Faculty of Mechanical Engineering, Brno University of Technology, Brno, Czech Republic; ^5^ CEITEC – Central European Institute of Technology, Brno University of Technology, Brno, Czech Republic; ^6^ CEITEC – Central European Institute of Technology, Masaryk University, Brno, Czech Republic; ^7^ Institute of Experimental Biology, Faculty of Science, Masaryk University, Brno, Czech Republic; ^8^ Department of Chemistry and Toxicology, Veterinary Research Institute, Brno, Czech Republic; ^9^ Department of Cytokinetics, Institute of Biophysics, Academy of Sciences of the Czech Republic, Brno, Czech Republic

**Keywords:** checkpoint kinase 1/Chk1, SCH900776, nucleoside analogs, chronic lymphocytic leukemia, TP53

## Abstract

Treatment options for *TP53*-mutated lymphoid tumors are very limited. In experimental models, *TP53*-mutated lymphomas were sensitive to direct inhibition of checkpoint kinase 1 (Chk1), a pivotal regulator of replication. We initially tested the potential of the highly specific Chk1 inhibitor SCH900776 to synergize with nucleoside analogs (NAs) fludarabine, cytarabine and gemcitabine in cell lines derived from B-cell malignancies. In p53-proficient NALM-6 cells, SCH900776 added to NAs enhanced signaling towards Chk1 (pSer317/pSer345), effectively blocked Chk1 activation (Ser296 autophosphorylation), increased replication stress (p53 and γ-H2AX accumulation) and temporarily potentiated apoptosis. In p53-defective MEC-1 cell line representing adverse chronic lymphocytic leukemia (CLL), Chk1 inhibition together with NAs led to enhanced and sustained replication stress and significantly potentiated apoptosis. Altogether, among 17 tested cell lines SCH900776 sensitized four of them to all three NAs. Focusing further on MEC-1 and co-treatment of SCH900776 with fludarabine, we disclosed chromosome pulverization in cells undergoing aberrant mitoses. SCH900776 also increased the effect of fludarabine in a proportion of primary CLL samples treated with pro-proliferative stimuli, including those with *TP53* disruption. Finally, we observed a fludarabine potentiation by SCH900776 in a T-cell leukemia 1 (*TCL1*)-driven mouse model of CLL. Collectively, we have substantiated the significant potential of Chk1 inhibition in B-lymphoid cells.

## INTRODUCTION

B-cell malignancies represent a heterogeneous group of lymphoproliferative diseases involving both rapidly growing and more indolent leukemia and lymphomas. Although mutations in the tumor-suppressor *TP53* are comparatively less frequent than in solid tumors, they are uniformly associated with an unfavorable outcome in respective patients [1]. For example, in chronic lymphocytic leukemia (CLL) *TP53* defects significantly reduce the efficacy of chemoimmunotherapy regimens functioning in other patients [2], and remain also challenging for innovative small-molecule inhibitors of B-cell receptor (BcR) signaling [3]. Recently, the inhibition of ataxia-telangiectasia mutated and Rad-3 related (ATR) kinase operating in the DNA damage response (DDR) pathway has been identified as a potential alternative therapeutic strategy in CLL [4]. ATR is a key molecule activating the DDR pathway through phosphorylation of checkpoint kinase 1 (Chk1) upon recognition of DNA damage [5, 6].

Chk1 represents a master cell cycle regulator which primarily supervises replication through intra-S and G2/M checkpoints and is also involved in other control points in G1 phase and mitosis [7]. The p53 protein integrates signals related to DNA damage, including the signaling mediated by Chk1, and consequently decides a cell's fate, principally between cell cycle arrest with DNA repair and apoptosis [8]. The Chk1 abrogation together with p53 inactivation can result in uncontrolled proliferation leading to direct apoptosis or mitotic catastrophe [9]. Whether the Chk1 inhibition can also be exploited for elimination of p53-wild-type (wt) cancer cells remains ambiguous. Some studies convincingly demonstrated a synergy between p53 deficiency and Chk1 inhibition [10, 11], but other more comprehensive approaches indicated that p53 status is only one of the decisive factors [12, 13].

Certain cell lines derived from lymphoid tumors display high sensitivity to direct (single agent) Chk1 inhibition [14], and this particularly concerns lymphoma cells in which c-Myc oncoprotein drives proliferation [15, 16]. These observations pose the question of whether Chk1 inhibition would be synergistic with DNA-damaging drugs specific for lymphoid cells. In fact, most recent studies analyzing Chk1-mediated sensitization to chemotherapy involved solid tumors or myeloid malignancies and used antimetabolites like hydroxyurea or gemcitabine (GEM) [17–19] with limited utility in the treatment of lymphoid tumors. By contrast, nucleoside analog fludarabine (FLU), a key chemotherapeutic for the most common leukemia, i.e. CLL, has been tested together with Chk1 inhibition only occasionally and the tests have been done in non-lymphoid cells [13, 20].

SCH900776 is a potent and highly selective Chk1 inhibitor identified through cell-based screening, in which accumulation of DNA double-strand breaks (DSBs) served as a functional readout [17]. The inhibitor had been selected as the functionally optimal compound with minimal antagonistic properties, and gemcitabine was later proposed to be an optimal chemotherapeutic partner. Of other nucleoside analogs (NAs), SCH900776 is also significantly synergistic with cytarabine (CYT) [17, 18, 21].

In our study, we initially analyzed the effects of Chk1 inhibition in combination with FLU, CYT, and GEM. These primarily S-phase specific NAs affect the cells through overlapping but not entirely congruent mechanisms of DNA damage induction [22]. The incorporation into replicating DNA is a common mechanism, whilst the inhibition of ribonucleotide reductase leading to a disturbed dNTP pool and incorporation to RNA are specific for GEM and FLU [23]. In addition, FLU is also able to affect non-dividing (quiescent) cells by interfering with DNA excision repair processes and initiating apoptosis through direct activation of apoptosome [23, 24]. We demonstrate that SCH900776 synergized with the tested NAs in a significant proportion of B-cell lines, primarily those with *TP53* gene disruption. Cell death mechanisms involved among others aberrant mitoses. Additionally, we demonstrate the effectiveness of SCH900776 with FLU in *TP53*-mutated primary CLL cells as well as in a wt-*TP53* T-cell leukemia 1 (*TCL-1*)-driven mouse model of human CLL.

## RESULTS

### SCH900776 blocks Chk1 activity in co-treatments with NAs

**Figure 1:**
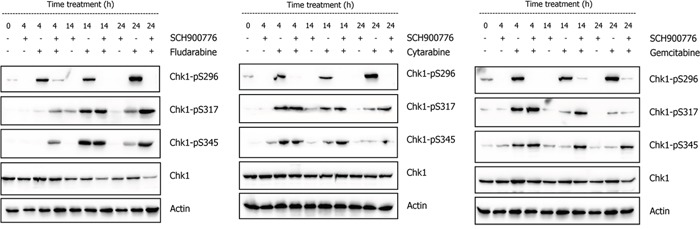
Effect of SCH900776 on Chk1 during the response of NALM-6 cell line to DNA damage Western blot analysis shows that administration of FLU (1 μg/ml), CYT (10 ng/ml) or GEM (5 ng/ml) activated Chk1 protein on serine 296 (pS296). This autophosphorylation was effectively blocked by SCH900776 (600 nM) in all tested intervals. Phosphorylations reflecting signaling of DNA damage and/or stalled replication forks towards Chk1 protein (pS317 and pS345) were potentiated by SCH900776 in the following intervals: FLU 24 h; CYT 14 h and 24 h; GEM 14 and 24 h. Total Chk1 protein level was reduced in FLU/SCH900776 co-treatment, which was apparent at 14 h and 24 h.

In order to gain insight on SCH900776 interference in DDR, we first analyzed its impact on Chk1 protein using NALM-6 cell line with wt-p53 status and baseline Chk1 autophosphorylation on Ser-296 (pS296; Chk1 activation marker). After 2 h pretreatment with DMSO (mock control) or 600 nM SCH900776, cells were incubated for 4 h, 14 h and 24 h with FLU, CYT, or GEM. As depicted in Figure 1, SCH900776 effectively suppressed Chk1 activation, both in presence and absence of NAs up until the last analyzed interval of 24 h.

At the same time, SCH900776 enhanced activating signaling towards Chk1 protein (phosphorylations pS317 and pS345) after cell exposure to individual NAs (Figure 1). Kinetics of both phosphorylations differed among cytostatics with a later onset after the FLU administration. In SCH900776 co-treatments with all three individual drugs, both phosphorylations persisted longer (up to 24 h) compared to treatments with NAs on their own. In the case of FLU, phosphorylations also occurred earlier during the respective co-treatment. Altogether, these results indicated distinct FLU kinetics in DNA damage induction compared to CYT and GEM and demonstrated SCH900776 effectiveness by blocking Chk1 activity.

### Chk1 inhibition augments p53 pathway activation and facilitates DNA DSBs formation in co-treatments with NAs

**Figure 2:**
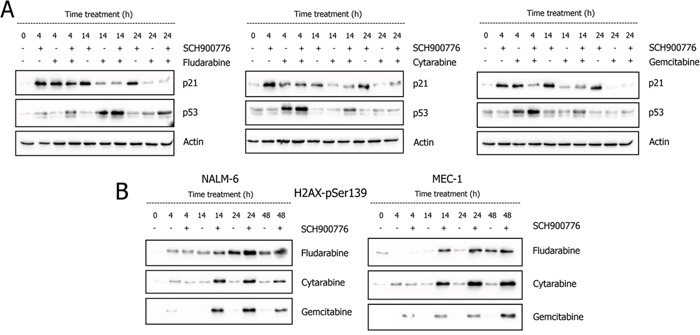
Effect of Chk1 inhibition on accumulation of p53 and p21 proteins A. and appearance of γ-H2AX B In wt-p53 NALM-6 cell line, the administration of SCH900776 (600 nM) led to a rapid (4 h) accumulation of p21, which persisted up to 24 h (boxes in individual cytostatics show the results of three independent experiments). Co-administration of NAs (concentrations as in Figure 1) then diminished or eliminated this effect. In contrast, NAs induced p53 protein, and this effect was augmented in Chk1-inhibited cells reaching its maximum at 4 h (CYT and GEM) or 14 h (FLU). The analysis of stalled replication and/or DNA DSBs accumulation (B), visualized by WB as phosphorylated histone H2AX at Ser139 (γ-H2AX) showed similar profile in NALM-6 and MEC-1 cell lines. In single agent treatments, only FLU elicited apparent γ-H2AX accumulation, whilst the signal was massive in all co-treatments with SCH900776. The NAs concentrations were the same as in Figure 1 for NALM-6 cell line and were the following for MEC-1 cells: FLU 10 μg/ml; CYT 1.6 μg/ml, and GEM 10 ng/ml.

To verify our assumption that SCH900776 increased DNA damage and replication stress during co-treatment with NAs [25], we initially analyzed p53 pathway activation in NALM-6 cell line. The p53 protein activates a G1-checkpoint after genotoxic stress, primarily through transcriptional activation of downstream genes including *CDKN1A* (coding for the p21 protein) [26]. Chk1 inhibition alone had no effect on the p53 level, while all three NAs elicited clear p53 stabilization with maximum induction coinciding with Chk1 activation; p53 accumulation was then further strengthened in NAs co-treatments with SCH900776 (Figure 2A).

SCH900776 alone strongly induced p21 protein, detectable from 4 h up to 24 h (Figure 2A). The p21 protein accumulation was also apparent but lower among treatments involving NAs (with or without SCH900776), which probably results from a proteosomal mediated p21 degradation reported in connection to all three NAs [27].

Real-time PCR analysis (Supplementary Figure S1A) then recorded negligible *CDKN1A* gene induction after treatment with SCH900776 on its own, which supports the view that the aforementioned p21 protein accumulation is p53-independent, and likely reflects a compensation for abrogated Chk1 activity through stabilized p21 protein [28]. By contrast, we observed an induction of p53 target genes *CDKN1A*, *BBC3* (PUMA), *BAX*, and *GADD45A* in treatments involving NAs (with or without Chk1 inhibitor). As expected, the MEC-1 cell line used further as a model of p53-mutated lymphoid tumor showed little or no induction of analyzed genes in tested combinations (Supplementary Figure S1B).

Conversion of DNA damage to DSBs is critical for activity of many anti-cancer agents, and Chk1 inhibition probably facilitates DSBs formation when stalled replication forks transform into replication forks collapse [28, 29]. We therefore analyzed histone H2AX phosphorylation on Ser-139 (γH2AX) in NALM-6 cells and MEC-1 cells (Figure 2B). Only FLU caused clear γH2AX accumulation as a single agent in NALM-6 cell line, and SCH900776 enhanced this process. In the case of CYT and GEM, markedly increased γH2AX levels were only present in co-treatments with SCH900776. In MEC-1 cell line, γH2AX accumulation exhibited a similar profile with a later onset after FLU treatment (48 h) compared to NALM-6 (24 h). Analyses thus confirmed that FLU affects DNA distinctly in comparison with CYT and GEM. In both cell lines, SCH900776 alone had no detectable effect on γH2AX level (data not shown).

### Chk1 inhibition significantly augments apoptosis in both p53-wt and p53-mutated cells

**Figure 3:**
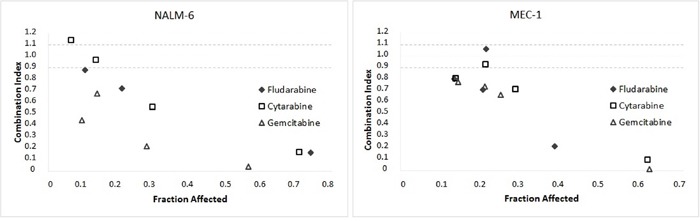
Apoptosis potentiation upon co-administration of SCH900776 with nucleoside analogs The graphs for NALM-6 and MEC-1 cell lines visualize apoptosis potentiation by SCH900776 (600 nM). Apoptosis was detected by flow cytometry analysis based on Annexin-V/PI staining. The analysis covers time points 4 h, 14 h, 24 h, and 48 h. In p53-wt NALM-6 cell line, all NAs elicited extensive cell death, which was further enhanced by the Chk1 inhibitor. In p53-mut MEC-1 cell line, a massive apoptosis was present only in the Chk1-inhibited cells. Combination indices (CI) were calculated according to the median-effect principle. Each point of the graph shows an affected fraction expressed relative to the untreated controls. CI < 0.9 represents synergism, CI 0.9-1.1 additive effect and CI > 1.1 antagonism. Table 1 summarizes the values for each measurement.

Using flow cytometry detection of Annexin-V/PI staining, we observed that in NALM-6 cell line FLU and CYT strongly induced apoptosis while GEM had only mild impact (Supplementary Figure S2A), and SCH900776 synergistically affected all three NAs (Figure 3 and Table 1). Apoptosis potentiation by SCH900776 was also confirmed by a cleavage of PARP (poly(ADP-ribose)polymerase) protein and a main effector caspase-3 (Supplementary Figure S2C). The p53-mutated MEC-1 cell line responded poorly to single agent treatments with NAs; however, the Chk1 inhibition in this drug resistant cell line significantly increased the proportion of apoptotic cells (Supplementary Figure S2B) manifesting synergy with the NAs (Figure 3 and Table 1). Notably, only a negligible fraction of cells stained Annexin-V negative and PI positive confirming apoptosis as a primary cell death mechanism.

**Table 1:** Effect of SCH900776 on apoptosis potentiation in NALM-6 and MEC-1 cell lines treated with nucleoside analogs

Cell line	Cytostatics	Dose (μg/ml)	Time (h)	Fraction Affected	Combination Index	Synergism
**NALM-6**	Fludarabine	1	4	0.05	1.411	--
			14	0.10	0.880	+
			24	0.21	0.720	++
			48	0.74	0.164	++++
	Cytarabine	0.01	4	0.06	1.140	-
			14	0.13	0.973	±
			24	0.29	0.561	+++
			48	0.71	0.164	++++
	Gemcitabine	0.005	4	0.09	0.445	+++
			14	0.14	0.675	+++
			24	0.28	0.216	++++
			48	0.56	0.042	+++++
**MEC-1**	Fludarabine	10	4	0.13	0.798	++
			14	0.20	0.707	++
			24	0.21	1.063	±
			48	0.38	0.216	++++
	Cytarabine	0.02	4	0.14	0.808	++
			14	0.21	0.927	±
			24	0.28	0.711	++
			48	0.61	0.095	+++++
	Gemcitabine	0.01	4	0.14	0.775	++
			14	0.21	0.734	++
			24	0.25	0.662	+++
			48	0.62	0.015	+++++

The ranges of CI and the symbols are refined from Chou-Talalay test.

Very strong synergism +++++; Strong synergism ++++; Synergism +++; Moderate synergism ++; Slight synergism +; Nearly additive ±; Slight antagonism -, Moderate antagonism –

**Figure 4:**
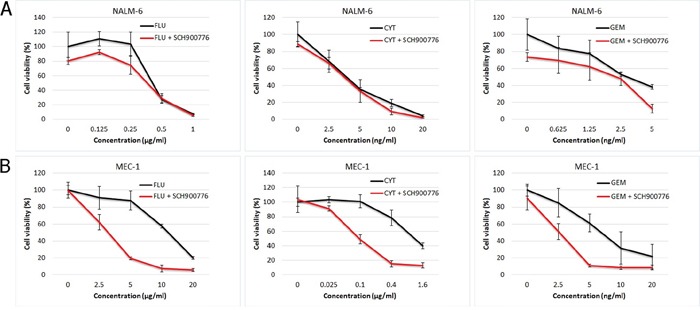
Effect of SCH900776 on viability of NALM-6 and MEC-1 cell lines treated with nucleoside analogs The cells were pre-treated for 2 h with SCH900776 (200 nM) and then cultured for 72 h in the presence of NAs using concentrations stated in the graphs. The cell viability was assessed by the WST-1 reagent. Chk1 inhibiton resulted in highly significant synergy with all three NAs in p53-mutated MEC-1 cells (P < 0.001), while this effect was non-significant in p53-wt NALM-6 cell line.

Finally, we assessed the impact of SCH900776 with NAs on overall viability of NALM-6 and MEC-1 cell lines. After a 72 h treatment, sensitive p53-wt cells revealed no effect of Chk1 inhibition, while p53-mutated cells were clearly sensitized by SCH900776 to all three NAs (Figure 4). To verify that this effect is indeed a consequence of Chk1 inhibition and not caused (in part) by compound-specific off-target effects, we used also a structurally different Chk1 inhibitor, CHIR-124 [30]. The analysis confirmed a clear sensitization of MEC-1 cell line to all three NAs (Supplementary Figure S3).

### Chk1 inhibition distinctly interfered with cell cycle profile in combination with individual NAs

Increased apoptosis in Chk1-inhibited cells could result from cell cycle changes if SCH900776 abrogates certain checkpoints activated by DNA-damaging drugs. The cell cycle analysis in NALM-6 cells (Supplementary Figure S4 and Supplementary Table S1) revealed that all three NAs administered with or without SCH900776 initially elicited G1-phase arrest. Cells released from the G1 arrest later accumulated in S-phase (maximum peak for GEM and CYT after 24 h and for FLU after 48 h), with the most pronounced accumulation observed for GEM. Chk1 inhibitor then affected these S-phase accumulated cells and finally, after 48 h, apoptotic fraction (sub-G1 peak) appeared. Intriguingly, the interval at 48 h indicated return to a regular cell cycle profile among CYT and GEM treatments, suggesting a vital phenotype rescue in a proportion of cells.

MEC-1 cell line analysis (Supplementary Figure S5 and Supplementary Table S2) then did not convey any G1 arrest after NAs treatments. Nevertheless, all three drugs were capable of inducing at least partial S-phase arrest (GEM and CYT (14 h); FLU (48 h)). The Chk1 inhibitor then presumably facilitated a quicker transit through the cell cycle, which resulted in a considerable sub-G1 fraction increase at the expense of S and G2/M populations. In contrast to NALM-6, we observed no restoration of normal cell cycle distribution. SCH900776 alone (600 nM) did not change cell cycle profile in the tested cell lines.

### Chk1 inhibition causes aberrant mitoses in MEC-1 cells treated with fludarabine

We hypothesized that cells with abrogated Chk1 activity might also die during mitosis considering the lack of Chk1 function in cell division [31]. Using a mitotic cell cycle arrest by colchicine and the analysis of chromosomes, we observed their normal appearance in NALM-6 cell line treated with FLU and SCH900776 (Supplementary Figure S6A-B). In evident contrast, we recorded aberrant mitoses with apparent chromosomal fragmentation (pulverization) with the same co-treatment in MEC-1 cells (Supplementary Figure S6C-D). This phenomenon was present in 18 out of 43 (42%) analyzed mitoses in co-treated cells, while it was only occasionally observed in single agent FLU (2 out of 52 mitoses; 4%), and never present in control cells (50 mitoses).

To obtain a direct insight on aberrant cell division indicated by cytogenetic analysis, we used the coherence-controlled holographic microscopy (CCHM) method [32] permitting time-lapse measurement of cell activity and mass changes with greater accuracy than other techniques. Untreated MEC-1 cells manifested homogeneously appearing mitoses with well-recognized separation of sister chromatids and the time course usually being 20-22 minutes (Supplementary Movie S1). By contrast, the cells treated with FLU and SCH900776 exhibited several abnormal features including (a) hardly recognizable metaphase plate, (b) inner cell mass asymmetric division between daughter cells, (c) accelerated time course of division process lasting approximately 14 minutes, and (d) signs of apoptosis such as membrane blebbing (Supplementary Movie S2).

### Chk1 inhibition significantly synergizes with NAs in other TP53-mutated lymphoid cell lines

We subsequently assessed the potential of Chk1 inhibition to synergize with FLU, CYT, and GEM in 17 B-lymphoma or B-leukemia cell lines; 10 of them harbored *TP53* disruption (Supplementary Table S3). In this experiment, we used 200 nM concentration of SCH900776 and the NAs concentrations determined individually for each cell line in order to achieve concentration-dependent viability curves. Generally, mut-p53 cell lines were more resistant to all three NAs than wt-p53 cells.

Concerning cell line proportion with synergistic effects, we recorded best results for GEM, followed by CYT and FLU (Table 2 and Supplementary Figure S7A-D). The combination treatment of SCH900776 with GEM exhibited synergy in 9 out of 10 mut-p53 cell lines, while, with CYT and FLU, synergism was registered in 5 and 4 out of 10 cell lines, respectively. Besides MEC-1, three other cell lines, i.e. SU-DHL-4, RAJI, and NALM-16, displayed synergy between SCH900776 and all three NAs. By contrast, in wt-p53 cell lines, SCH900776 enhanced the cytotoxicity only in 1 out of 7 (CYT), 2 out of 7 (FLU), and 3 out of 7 cases (GEM). No wt-p53 cell line then showed synergistic effect in all three NAs.

**Table 2:** Effect of SCH900776 on overall viability of B-lymphoid cell lines treated with nucleoside analogs

Cell lines	FLU (μg/ml)	Synergism	CYT (ng/ml)	Synergism	GEM (ng/ml)	Synergism
**MEC-1**	2.5-20	+++	25-1600	+++	2.5-20	+++
**MEC-2**	2.5-20	ns	2500-20000	++	0.4-25	+++
**SU-DHL-4**	2.5-20	+++	25-1600	+++	0.4-25	+++
**RAJI**	4-10	+++	25-1600	+++	2.5-20	+++
**BL-41**	2.5-20	ns	1.6-100	ns	0.625-5	+++
**RAMOS**	0.25-2	ns	12.5-100	ns	0.625-5	+++
**JEKO-1**	0.125-1	ns	2.5-20	ns	0.625-5	+++
**REC-1**	2.5-20	ns	4-250	ns	0.25-2	ns
**REH**	0.125-1	ns	2.5-20	ns	1.25-10	++
**NALM-16**	0.25-2	+	2.5-20	++	0.625-5	+++
**NALM-6**	0.125-1	ns	2.5-20	ns	0.625-5	ns
**JVM-2**	0.25-2	ns	25-1600	ns	0.625-5	+++
**JVM-3**	2.5-20	++	25-200	++	0.313-2.5	ns
**WSU-NHL**	0.125-1	ns	0.4-25	ns	0.25-2	+
**DOHH-2**	0.25-2	ns	1.6-100	ns	0.25-2	ns
**GRANTA-452**	0.125-1	ns	2.5-20	ns	0.625-5	ns
**GRANTA-519**	0.25-2	++	1.6-100	ns	0.625-5	+++

The cells were treated with four concentrations of NAs; a concentration range is stated for each cell line and NA. Statistical significance of synergistic effects was assessed by ANOVA test; + P < 0.05; ++ P < 0.01; +++ P < 0.001. The cell lines represent chronic lymphocytic leukemia in prolymphocytoid transformation (MEC-1, MEC-2; derived from the same patient with ~1 year interval between the samplings), diffuse large B cell lymphoma (SU-DHL-4, WSU-NHL), Burkitt lymphoma (RAJI, BL-41, RAMOS), mantle cell lymphoma (JEKO-1, REC-1, GRANTA-519), acute lymphoblastic leukemia (REH, NALM-16, NALM-6, GRANTA-452), prolymphocytic leukemia (JVM-2, JVM-3), and follicular lymphoma (DOHH-2).

### Chk1 inhibition potentiates fludarabine in primary CLL cells treated with pro-proliferative stimuli

Next, we analyzed the potential of SCH900776 to synergize with FLU in primary CLL cells. Since CLL lymphocytes from peripheral blood are in a resting stage, we had to apply pro-proliferative stimuli to induce a cell cycle with presumable Chk1 activity [4]. We co-cultured CLL cells with the CD32-transfected murine L-cells, anti-CD40 antibody, and IL-4, because such a system mimics the microenvironment supporting CLL cell proliferation [33]. This stimulation resulted in CLL cell enlargement (Supplementary Figure S8A) resembling blastoid transformation and cell cycle entrance, as demonstrated by increased expression of *Ki67* and *AID* (activation-induced cytidine deaminase) genes [33] (Table 3), and Ki67 protein (Supplementary Figure S8B). Furthermore, a proportion of CLL cells underwent active division, as evidenced by the cell cycle analysis (Supplementary Figure S8C) and CFSE dilution staining [4] (Supplementary Figure S8D). CLL cell stimulation also resulted in changes of pro-proliferative (c-Myc, STAT3) and apoptosis-regulatory (Bcl-2) proteins and, most importantly also in a clear accumulation of activated Chk1 protein in CLL cells (Supplementary Figure S9).

**Table 3:** Synergy of SCH900776 with fludarabine in primary CLL cells

Sample	*TP53* mutation (% of affected clone)	Defects by FISH	Response to FLU^a^	Previous therapy	Ki67 RNA^#^	AID RNA^#^	Synergism^b^
**CLL1**	p.T155I (52%) p.R158H (20.5%) p.R273C (7%) c.375+1G>A (11.6%) c.783-1G>A (5.7%)	13q- (9%)	Resistant	FC, FCR, A, R+D, R+CLB	19.3	4648	+++/ns
**CLL2**	p.Q317* (98.2%)	17p- (95%) 13q- (52%)	Resistant	no	70	8582	+++/ns
**CLL3**	p.F113del (50%)	13q- (89%)	Sensitive	R+D, FCR	132	96	++/ns
**CLL4**	p.Y107* (98.2%)	17p- (86%) +12 (6%)	Resistant	no	9.7	5.9	+/ns
**CLL5**	c.942_955del (99.9%)	17p- (95%) +12 (20%)	Resistant	multiple	n.a.	n.a.	ns/n.a.
**CLL6**	p.R282W (99.9%)	13q- (97%)	Resistant	FCR, A	275	189	ns/n.a.
**CLL7**	c.816_841del (95%)	17p- (83%)	Resistant	no	6.5	1516	ns/n.a.
**CLL8**	c.626_627del (62.8%)	13q- (80%)	Resistant	no	n.a.	n.a.	ns/n.a.
**CLL9**	no	No	Resistant	FCR, A, O+D	n.a.	n.a.	+/ns
**CLL10**	no	no	Sensitive	FCR	2.6	242	ns/n.a.
**CLL11**	no	11q- (70%)	Sensitive	no	n.a.	n.a.	ns/n.a.
**CLL12**	no	13q- (94%)	Sensitive	no	n.a.	n.a.	ns/n.a.

# Fold change detected by real-time PCR compares gene expression of CLL cells treated with pro-proliferative stimuli for 6 days to the non-stimulated CLL cells (harvested before the stimulation on day 0). Murine L-cells used in the co-culture system did not show any specific amplification of *Ki67* and *AID* genes.

a Resistant: viability >50% at the highest concentration of fludarabine in stimulated cells; Sensitive: viability < 50%.

b CLL cells treated with pro-proliferative stimuli/non-stimulated CLL cells. ns: not significant; n.a.: not analyzed

FC: fludarabine, cyclophosphamid; FCR: FC with rituximab; A: alemtuzumab; R+D: rituximab with dexamethasone; R+CLB: rituximab with chlorambucil; O+D: ofatumumab with dexamethasone

**Figure 5:**
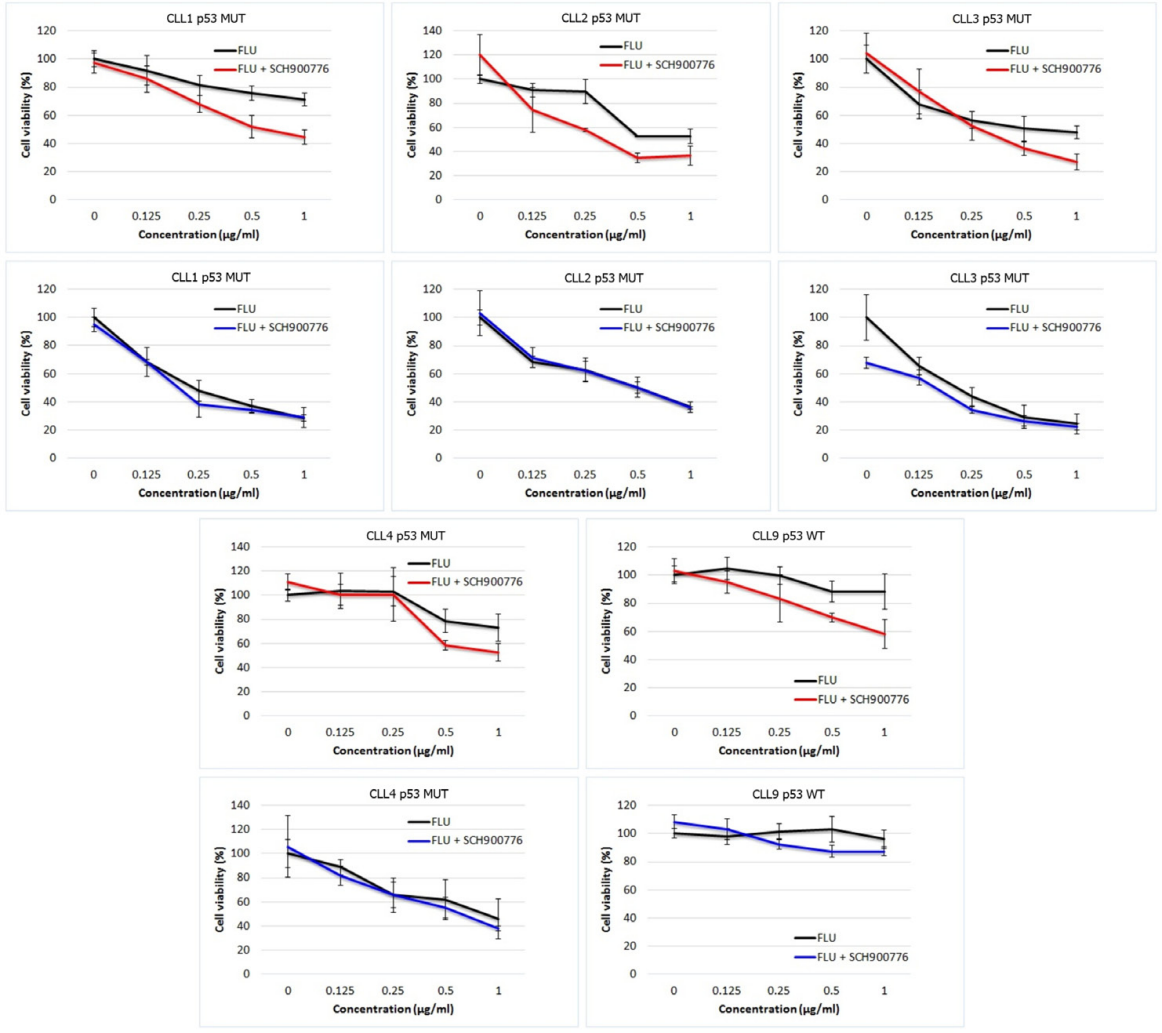
Effects of Chk1 inhibition in stimulated and non-stimulated CLL cells The common treatment of SCH900776 with FLU resulted in the significant synergistic effects in CLL cells pre-treated with the pro-proliferative stimuli (red curves), while this co-treatment did not provide any advantage over single agent FLU in non-stimulated CLL cells (blue curves). The statistical significances for individual samples are listed in Table 3. The graphs show cultures with positive effect of Chk1 inhibition in stimulated cells.

When we applied SCH900776 and FLU as in the permanent cell lines (i.e. the inhibitor added 2 h before FLU), we observed no sensitization effects (data not shown). We hypothesized that SCH900776 activity possibly diminished due to the enhanced anti-apoptotic protection accompanying CLL cell activation [34]. We therefore maximized the impact of Chk1 inhibition by initially inducing DNA damage (18 h cultivation with FLU) followed by later inhibition of activated Chk1 through SCH900776 administration [19, 35]. Indeed, this modified schedule resulted in the synergistic effect of SCH900776 with FLU in 4 out of 8 *TP53*-mutated samples (Table 3 and Figure 5). The wt-*TP53* samples were typically sensitive to FLU and thus Chk1 inhibition did not provide additional effect. However, one resistant sample (CLL9 in Table 3 and Figure 5) revealed synergy between the Chk1 inhibition and FLU. As anticipated, we did not observe any positive effects of SCH900776 in non-stimulated CLL cells, which have only residual Chk1 activity (Table 3 and Figure 5).

### Chk1 inhibition potentiates fludarabine in Eμ-TCL1 mouse model of CLL

**Figure 6:**
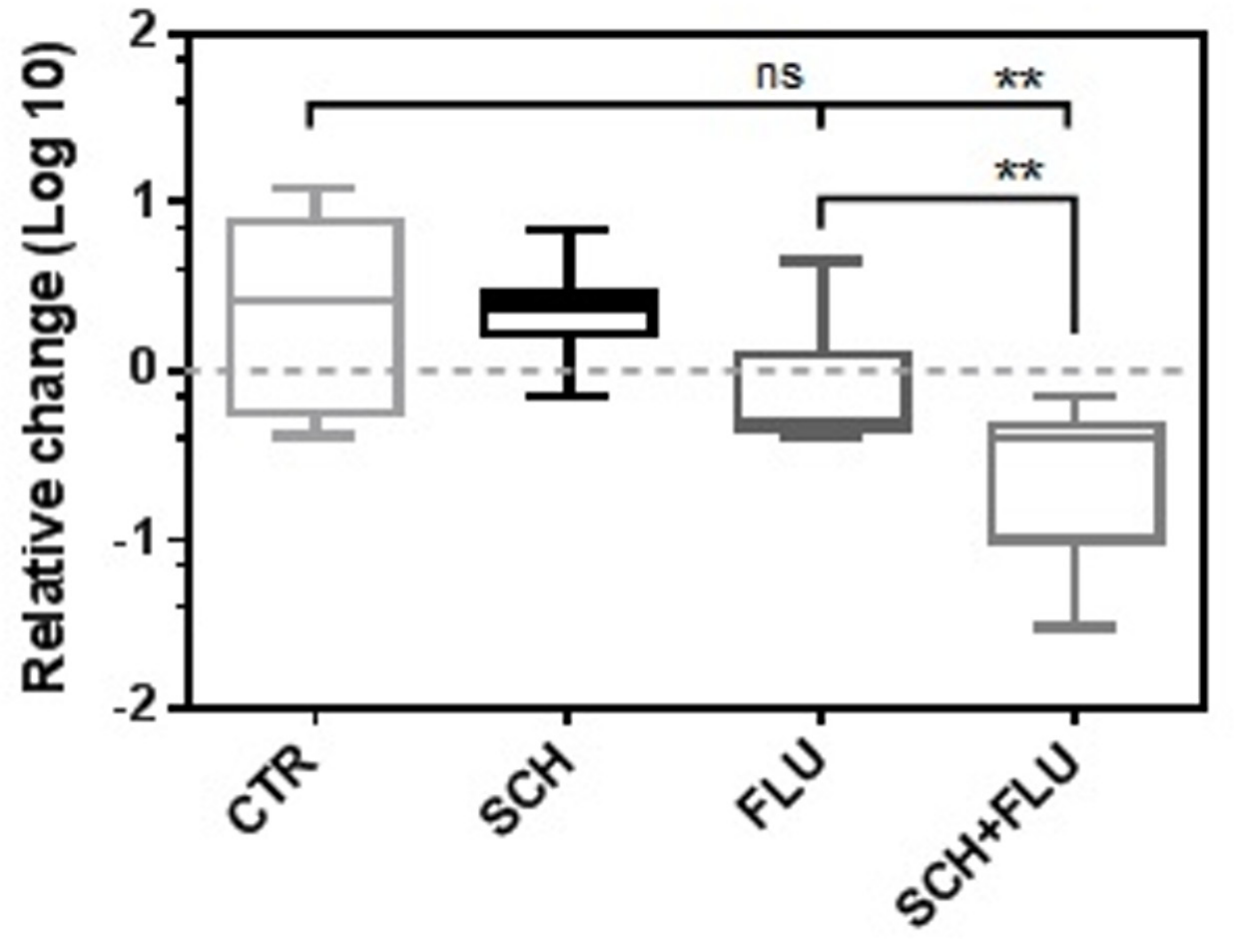
Effects of SCH900776 and FLU in the mouse Eμ-TCL1 model of CLL The 5-day treatment involved daily intraperitoneal administrations of SCH900776 (N = 7 mice), FLU (N = 9 mice), SCH900776 with FLU (N = 10 mice) or control 20% kolliphor/PBS solution (N = 6 mice). The effects are presented as relative change of leukemic cell counts in peripheral blood detected in the samplings after the treatment compared to initial cell counts (1 = no change). FLU administered together with the Chk1 inhibitor had superior effect compared to FLU as a single agent (Mann-Whitney test, P = 0.0071) and untreated control mice (P = 0.0100). SCH900776 as a single agent did not manifest a cytotoxic effect.

Mice transgenic for the *TCL1* gene under the control of B-cell specific promoter (Eμ-TCL1 mice) develop a disease plausibly resembling human CLL [36]. This model provides the specific phenotype of leukemic cells (B220^dim^CD5^+^), which enables relatively easy assessment of the disease progression and/or efficacy of anti-cancer drugs. The effects of SCH900776 and FLU as single agents, and in combination, achieved within our 5-day treatment (daily intraperitoneal administrations) are summarized in Figure 6 and Supplementary Figure S10. The effects of FLU applied together with the Chk1 inhibitor were superior in comparison with both single agents FLU and SCH900776.

## DISCUSSION

Chk1 inhibitors represent particularly promising therapeutics enhancing anti-cancer activity of DNA-damaging drugs [7, 11, 13, 17, 37]. We analyzed this potential in B-cell leukemia and lymphoma cells using one of the most specific Chk1 inhibitors SCH900776. In order to assess its kinase selectivity, we profiled the inhibitor in a radiometric *in vitro* assay against 207 human kinases (Merck Millipore KinaseProfiler, now Eurofins). SCH900776 used at 1 μM concentration inhibited only 12 kinases with efficacy that corresponded to the residual kinase activity lower than 30% (Supplementary Table S4). According to the comprehensive *UniProt* database (www.uniprot.org) only TLK2 and Rsk4 from the list of the inhibited kinases were directly involved in DDR. Of note, the selectivity profile of SCH900776 and its efficacy in cell-based assays fulfill the criteria postulated for a “quality kinase probe” [38].

We initially analyzed Chk1 inhibition effects in wt-p53 and mut-p53 cell lines, and besides CYT and GEM representing well-established NAs suitable for synergistic Chk1 inhibition, we analyzed the effect of FLU as a drug specific for B-lymphoid tumors. In the p53-proficient cell line, all three NAs elicited signaling towards Chk1 (pS317, pS345), which was further intensified in co-treatments with SCH900776; in all three NAs the inhibitor reliably abrogated autophosphorylation at Ser296 reflecting actual Chk1 activity [18, 37]. The attempt of a cell to activate Chk1 coincided with p53 accumulation further reflecting the response to DNA damage and/or replication stress [39]. Interestingly, p21 protein showed presumably dual induction: p53-independent upon administration of SCH900776 alone and p53-dependent in the treatments involving NAs, which probably reflects the complex interplay among Chk1, p53 and p21 proteins [40].

Concerning the critical event accompanying Chk1 inhibition, i.e. conversion of stalled replication forks to DNA DSBs [41], we observed both in NALM-6 and MEC-1 cell lines that (i) only FLU was able to induce significant amount of γ-H2AX as a single agent, and (ii) in all co-treatments of SCH900776 with the NAs, the γ-H2AX level was clearly increased. The γH2AX accumulation after FLU treatment can likely result from the inability to excise this NA from DNA during repair [25]. The analysis of apoptosis then affirmed its clear intensification through SCH900776 in the case of all three NAs. This supports the view that excessive DSBs originating after replication forks collapse represent to cells the critical type of DNA damage [42].

Due to the pre-treatment with SCH900776, all three primarily S-phase-specific NAs entered the cells with abrogated Chk1 activity thus impacting both replication forks stability [43] and cell cycle control [44]. In the cell cycle analysis, all three NAs manifested similar effects including (i) an induction of a prominent G1-arrest in p53-wt cells (ii) the later cell accumulation in S-phase regardless of the p53 functional status, and (iii) final increase of apoptotic sub-G1 fraction at the expense of S-phase accumulated cells.

Concerning a more exact timing of cell death, we assumed that in addition to direct apoptosis initiated in the S-phase [20, 45, 46] some cells might proceed further through the cycle and undergo mitotic catastrophe [47, 48], an event which could also reflect Chk1 role in the chromosome segregation and cytokinesis [49]. After the FLU/SCH900776 co-treatment, we observed severely damaged chromosomes in p53-mutated MEC-1 cells but not in p53-wt NALM-6 cells, confirming partially distinct cell death mechanisms in these two cell lines. The time-lapse live-cell imaging then confirmed the aberrant course of mitoses in MEC-1 cells. In total, the differences between the MEC-1 cells treated with FLU and FLU with SCH900776 were detectable throughout the cell cycle from S-phase to mitosis.

In the last experiment comparing tested NAs, we analyzed their synergy with SCH900776 in 17 B-cell lines. We observed that (i) GEM was superior in terms of sensitized cell lines number confirming its position of optimal partner drug for SCH900776 [17] also in leukemia and lymphoma cells. Although GEM seems to be optimal from the biological point of view, its clinical utility is limited in some cancers due to the excessive toxicity or weaker efficacy compared to other drugs. In CLL, the use of FLU clearly predominates. Recently, GEM has nevertheless been reported to be active in *TP53*-aberrant or relapsed/refractory CLL [50], and a similar observation has also been published for CYT, including its effect in highly adverse Richter syndrome (CLL transformation to diffuse large B cell lymphoma) [51]. Our study thus further supports the potential utility of all three NAs in CLL by demonstrating their synergy with Chk1 inhibition.

In order to identify potential determinants of cell sensitization through Chk1 inhibition, we analyzed in our cell lines the baseline level of the following proteins: c-Myc, Bcl-2, Mcl-1, NF-κB, and Chk1 itself (Supplementary Figure S11). The up-regulated *c-MYC* expression was proposed to be a significant contributor to increased replication stress [42], and we observed higher c-Myc levels in all cell lines harboring its translocation, i.e. RAJI, BL-41, RAMOS, DOHH-2, and GRANTA-452 (Supplementary Table S3) and, in addition, in NALM-16 and REC-1 cells. However, the c-Myc level alone did not predict a universal synergy between the Chk1 inhibition and all three NAs. We also noted a lack of such association for the other analyzed proteins; most cell lines either exhibited higher Bcl-2 or Mcl-1 level probably reflecting an anti-apoptotic protection through one of these two proteins. Finally, our mutational analysis of the *ATM* gene, a key mediator of cellular response to DSBs [26, 42] revealed only one *ATM*-mutated cell line in our panel, previously reported GRANTA-519. Therefore, a marker predicting synergy between Chk1 inhibition and tested NAs remains currently elusive which likely reflects the heterogeneous set of B-cell lines with diverse genetic background.

We also demonstrated that SCH900776 potentiates FLU in primary CLL samples. Pathogenesis of this disease involves (i) BcR stimulation by an antigen or autoantigen, (ii) presence of intrinsic genetic defects, and (iii) support by microenvironment stimuli [52]. Due to this complexity, CLL lymphocytes withdrawn from patient's peripheral blood do not proliferate and, without protection, die in tissue culture by apoptosis. Nevertheless, the employed co-culture system providing pro-proliferative signaling [33, 34] allowed both prolonged CLL cell survival and partial proliferation, thus constituting a relevant environment for Chk1 inhibition testing. Results of our tests revealed a potentiation of FLU through SCH900776 in half of stimulated *TP53*-mutated samples. This synergy was also effective in *TP53*-wt CLL cells resistant to FLU. Our study thus supports the potential clinical utility of ATR/Chk1 pathway inhibition in CLL, thereby adding Chk1 to the previously suggested target ATR [4].

To address the question of SCH900776 activity *in vivo*, we took advantage of the Eμ-TCL1transgenic mice, so far considered to be one of the best *in vivo* models for this disease, following its natural development and covering all major symptoms present in human CLL [36]. In previous study [53], FLU alone has been shown to mildly prolong survival of Eμ-TCL1 mice during the long-term treatment, and has exhibited an *in vitro* activity against leukemic cells derived from these mice. In our study, relatively weak FLU effects in the short-term experiment were substantially improved by the addition of Chk1 inhibitor. Importantly, Eμ-TCL1 mice have been shown to harbor wt-*TP53* gene [53]; our results thus demonstrate the positive impact of Chk1 inhibition during FLU treatment in this setting.

In conclusion, our report demonstrates that Chk1 inhibition can significantly surmount the intrinsic resistance of B-lymphoid cancer cells to NAs. By our pilot study, we provide evidence that SCH900776 potentiates FLU activity in currently incurable CLL and that this effect is achievable in both *TP53*-mutated and *TP53*-wt cells. Further testing of the clinical candidate SCH900776 and other specific Chk1 inhibitors in B-cell malignancies, including CLL, seems warranted and can be particularly appropriate for *TP53-*mutated or fludarabine-refractory patients.

## MATERIALS AND METHODS

### Cell lines and primary CLL cells

Cell lines representing B-cell malignancies were obtained from the German Collection of Microorganisms and Cell Cultures (DSMZ), and were cultured at 37°C and 5% CO_2_ in media from Biowest (IMDM) or Sigma-Aldrich (all other media) supplemented with fetal bovine serum (FBS) (MP Biomedicals). Individual cell lines were cultured in accordance with the DSMZ recommendations. CLL samples (peripheral blood mononuclear cells) were obtained from patients monitored or treated at the Department of Internal Medicine – Hematology and Oncology of the University Hospital Brno; written informed consent was available for all patients, and the study was approved by the Ethics Committee of the Faculty of Medicine of Masaryk University (project no. 15-33999A). A proportion of leukemic cells (CD5^+^/CD19^+^) exceeded 90%; non-stimulated cells were cultured in RPMI-1640 medium with 10% FBS.

We verified *TP53* mutation status in all cell lines by the yeast functional analysis (FASAY) coupled to sequencing [54]. The mutations corresponded to database of the International Agency for Research on Cancer (IARC) [55] or to the literature data except REC-1 cells reported as *TP53*-wt [56], but in fact harboring two mutations. The *TP53* gene in CLL samples was investigated by the next generation sequencing (NGS) using MiSeq instrument (Illumina) and conditions specified by us previously [57]. Cytogenetic aberrations were detected by fluorescence *in situ* hybridization (FISH) using probes from MetaSystems. The mutational analysis of *ATM* gene was performed in all cell lines by NGS on MiSeq; details are available upon request.

### Antibodies

Antibodies (Ab) against Chk1, Chk1-pSer296, Chk1-pSer317, Chk1-pSer345, p21, beta-Actin, H2AX, H2AX-pSer139, total PARP, cleaved-PARP, total caspase-3, cleaved-caspase-3, c-Myc, Bcl-2, Mcl-1, STAT3-pSer727, and STAT3-pTyr705 were purchased from Cell Signaling Technology. Anti-NFκB Ab was from Santa Cruz Biotechnology. Anti-p53 Ab (DO-1) was a gift from Dr. Borivoj Vojtesek (Masaryk Memorial Cancer Institute, Brno). Anti-rabbit and anti-mouse secondary antibodies were purchased from DakoCytomation. Anti-Ki-67-PE Ab and appropriate isotype control were purchased from BioLegend.

### Drugs

Cytarabine, gemcitabine, and fludarabine were purchased from Sigma-Aldrich. At the beginning of the project, Chk1 inhibitor SCH900776 (Merck; MK-8776) was not commercially available and was thus prepared in-house by a method based on previously published methodology [58, 59] and the newly discovered separation of the racemic target compound at the end of the synthesis (Supplementary Method S1). The inhibitor was dissolved in DMSO as 100 μM stock solution and stored at room temperature (RT).

### Western blot analysis

Cells were seeded in 6-well plates (5 × 10^6^ cells per well; volume 5 ml) and after treatment were lysed in ice-cold RIPA buffer with protease and phosphatase inhibitor cocktails (Sigma-Aldrich). Protein concentrations were determined by BCA Protein Assay Kit (Sigma-Aldrich). 5-100 μg of lysates were separated on 10-15% sodium dodecyl sulfate polyacrylamide gel (SDS-PAGE) and transferred to a nitrocellulose membrane. Membranes were hybridized with the primary antibodies overnight at 4°C or for 2 h at RT (DO-1). Proteins were visualized using Lumi-Light Western Blotting Substrate (Roche) or Clarity Western ECL Substrate (Bio-Rad) and analyzed by Alliance 4.7 software (Uvitec) or exposed on chemiluminiscent film.

### Real time PCR analysis

Cell lines were treated in 6-well plates (5 × 10^6^ cells per well; volume 5 ml). Real-time PCR was performed using TaqMan technology and 7900 Real Time PCR System (Applied Biosystems). Primer and probe set was specific for *CDKN1A*, *BBC3*, *BAX* and *GADD45A* genes (Applied Biosystems). In CLL cells, we used SensiFAST SYBR green intercalating dye (Labmark) and PCR primers specific for human *MKi67* and *AICDA* genes (primers available upon request). In both methodologies, we used 500 ng of RNA and SuperScript II reverse transcriptase (Thermo Scientific) according to manufacturer's instructions. The threshold cycle (Ct) values were exported to Microsoft Excel for 2^−ΔΔCt^ analysis. The geometric mean of *TBP* (TATA-box binding protein) and *HPRT* (hypoxanthine-guanine phosphoribosyltransferase) genes served as an internal standard.

### Cell cycle analysis

Cell lines were seeded in 6-well plates (5 × 10^6^ cells per well; volume 5 ml), pre-incubated for 2 h with 600 nM SCH900776, and subjected to treatment with NAs for 4 h, 14 h, 24 h and 48 h. Stimulated CLL cells were cultured as stated in “Stimulation and treatment of CLL cells”. The cells were harvested and fixed in ice-cold 70% ethanol and stored at −20°C. DNA content was analyzed by PI staining using a Cell Lab Quanta SC flow cytometer (Beckman Coulter) and Cell Lab QUANTA software (Beckman Coulter). 30,000 (cell lines) or 10,000 (CLL) cells were counted per sample.

### Apoptosis

We used Annexin V-APC/PI staining (Exbio) and flow cytometry detection (BD FACSVerse). Treated cells were harvested and stained according to the manufacturer's instructions. 30,000 cells were counted per sample.

### *In vitro* cytotoxicity assay

The cell lines were seeded in 96-well plates in quadruplicates (5 × 10^4^ cells per well, volume 200 μl); half of these cells was treated with 200 nM SCH900776, and after 2 h NAs were applied for 72 h. CLL cells were treated as stated in “Stimulation and treatment of CLL cells”. Four hours before the end of incubation, 10 μl of WST-1 Cell Proliferation Reagent (Roche) was added, and the absorbance was read at 450 nm using spectrophotometer 1420 Multilabel Counter Victor (PerkinElmer).

### Live-cell imaging

Cell death in MEC-1 cell line was visualized using holographic microscopy [32]. The cells were seeded in a recording chamber at the density of 2.5 × 10^5^/ml, cultured for 2 h with SCH900776 (200 nM) and then for 36 h with FLU (5 μg/ml) or for 36 h with FLU only. Thereafter, the cells were recorded for additional 30 h using time-lapse Multimodal Holographic Microscope Q-PHASE (IPE BUT, Tescan) [32]. This system based on an off-axis interferometer setup with an incoherent light source provides quantitative phase imaging (QPI) which enables quantitative measurement of cell mass [60].

### Cytogenetic analysis of mitotic cells

MEC-1 cells (2.5 × 10^5^/ml; volume 5 ml) were cultured with FLU for 48 h (5 μg/ml) with or without 2 h SCH900776 (200 nM) pre-treatment. NALM-6 cells were cultured the same way using FLU concentration 0.5 μg/ml. In both cell lines, the FLU/SCH900776 co-treatment caused similar effect on cell viability reduction (see Figure 4). Thereafter, the cells were arrested in mitosis by the additional 7 h (MEC-1) or 6 h (NALM-6) culture in the presence of colchicine (0.01%). The cell suspensions were fixed with Carnoy solution (methanol-acetic acid 3:1), pretreated with trypsin and stained with Giemsa-Romanowski solution according to the standard protocol [61]. The mitotic index reflecting a percentage of cells in mitosis was assessed by visual inspection using microscope Olympus BX 60 and evaluating 1,000 cells. The structure of mitotic chromosomes was assessed in 50 mitotic spreads identified using the system SKY (Applied Spectral Imaging) for karyotyping.

### Stimulation and treatment of CLL cells

CD32-transfected murine L-cells were a gift from Dr. Nicholas Chiorazzi (The Feinstein Institute for Medical Research, Manhasett, NY). The cells irradiated by 50 Gy were seeded in 6-well plates (5 × 10^5^ cells per well), and CLL cells were added on the next day at a ratio 30:1. Stimulation conditions using anti-CD40 Mab and IL-4 were as described in [33]. After six days, CLL cells were gently removed and cultured for 3 h to allow occasional L-cells to attach; the typical purity was over 98.5% of CD5^+^/CD19^+^ (CLL) cells. The cells were treated for 18 h with FLU (24-well plates) followed by co-treatment with SCH900776 (400 nM) or DMSO up to 72 h, in 96-well plates in quadruplicates. Non-stimulated CLL cells were treated with FLU and SCH900776 similarly to their stimulated counterparts.

### CFSE staining

Carboxyfluorescein diacetate succinimidyl ester (CFSE; Thermo Scientific) was used according to manufacturer's instructions to track CLL cell proliferation after stimulation. Briefly, 2 × 10^6^ cells were washed once with PBS and stained with 1.25 μM CFSE for 5 minutes in the dark. 20% cold FBS was added to quench the reaction. The cells were washed once with RPMI supplemented with 10% FBS before plating onto irradiated L cells.

### Animal experiments

Eμ-TCL1 transgenic mice [36] were initially obtained from Dr. Alexander Egle (Paracelsus Medical University, Salzburg, Austria). Mice were housed in a specific pathogen-free facility, given autoclaved food, and maintained on UV-sterilized water with 12 h light/dark cycles. Experiments were performed with the approval of the Ethics Committee of the Faculty of Medicine of Masaryk University. Eleven to thirteen-month-old mice that spontaneously developed CLL-like B-cell leukemia were randomized into groups with similar disease stage according to the white blood cell counts. Animals were treated for 5 days by intraperitoneal injection of: SCH900776 dissolved in 20% kolliphor solution (30 mg/kg/day; N=7 mice), fludarabine (DC Chemicals) dissolved in PBS (34 mg/kg/day, N=9 mice) or SCH900776 and fludarabine together (N=10 mice). Six control animals were injected with 20% kolliphor/PBS solution only. Before the experiment and after the 5-day treatment, peripheral blood was collected from a tail vain, and total leukemic cell counts were assessed by flow cytometry as described previously [62] using the following antibodies from eBioscience: anti-Mouse CD5-FITC (clone 53-7.3) and anti-Human/Mouse CD45R (B220)-PE-Cyanine5 (clone RA3-6B2). Anti-Mouse CD3-PE (clone 17A2) was used to eliminate T-cells. The leukemic cell detection is summarized in Supplementary Method S2. The B220^low^ CD5^+^ leukemic cell counts were compared between the control and treated animals. Accuri C6 flow cytometer (BD Bioscience) and Accuri C6 software were used to perform the analysis.

### Statistical analyses

WST-1 cell viability assay was evaluated by two-way analysis of variance (ANOVA), comparing the effect of NA vs. NA with SCH900776. The flow cytometry analysis analysis of apoptosis (Annexin-V/PI staining) was evaluated by Chou-Talalay test using CompuSyn software. In animal experiments, normality distribution was tested by the Kolmogorov–Smirnov, Shapiro–Wilk, or D'Agostino and Pearson tests. Accordingly, nonparametric tests were used to assess the differences between two variables (Mann–Whitney test) or the differences in the paired samples (Wilcoxon signed rank test). All tests were performed as two-sided using GraphPad Prism 5 software (GraphPad Software Inc.).

## SUPPLEMENTARY METHOD, FIGURES AND TABLES








