# Supplementary material for: Chk1 inhibition significantly potentiates activity of nucleoside analogs in TP53-mutated B-lymphoid cells
Source: Oncotarget. 2016 Aug 19;7(38):62091–106. doi: 10.18632/oncotarget.11388 (PMC5308713; doi:10.18632/oncotarget.11388)
Supplement: Supplementary file 1 [file oncotarget-07-62091-s001.pdf]

Chk1 inhibition significantly potentiates activity of nucleoside analogs in TP53-mutated B-lymphoid cells

SUPPLEMENTARY METHOD S1: SYNTHESIS OF INHIBITOR SCH900776[1]

General information

All chemicals were purchased from commercial suppliers and used without further purification. Anhydrous solvents were purchased as extra dry and stored over 4Å molecular sieves. Unless stated otherwise, the reactions were carried out in oven-dried glassware under atmosphere of nitrogen.

HRMS spectra were measured on Agilent 6224 Accurate-Mass TOF LC-MS with dual electrospray/chemical ionization mode with mass accuracy greater than 2 ppm. NMR spectra were recorded on Bruker Avance 300 MHz spectrometer. Chemical shifts (δ in ppm) of ^{1}H NMR and ^{13}C NMR are referenced to the residual signals of solvents: $CDCl_3$ [7.26 (^{1}H) and 77.23 (^{13}C)] and DMSO-d_6 [2.50 (^{1}H) and 39.51 (^{13}C)]. Data are represented as follows: chemical shift, multiplicity (s = singlet, d = doublet, t = triplet, q = quartet, m = multiplet and/or multiple resonances), coupling constant (*J*) in Hertz, integration. IR spectra (4000-400 cm-1) were collected on an EQUINOX 55/S/NIR FTIR spectrometer. Samples were prepared as KBr pellets. Column chromatography was carried on silica gel (230-400 mesh). TLC plates (silica gel 60 F_{254} or silica gel-RP18 60 F_{254}) were visualized under UV. HPLC separations were performed on Agilent 1260 Infinity LC system with UV detection using semipreparative columns with chiral stationary phase: Chiralcel® OJ™.

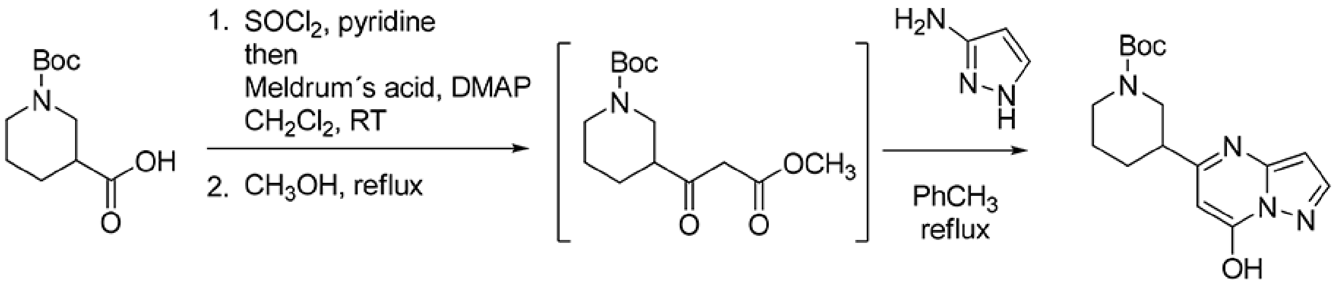

1 2 3

Compound 3

Thionyl chloride (1.9 mL, 26.2 mmol, 1.20 equiv) was added dropwise to a stirred solution of Boc-protected acid **1** (5.01 g, 21.9 mmol, 1 equiv) and pyridine (4.41 mL, 54.8 mmol, 2.50 equiv) in dichloromethane (30 mL) at room temperature. The resulting solution was stirred at this temperature for 30 min. Then, 4-dimethylaminopyridine (6.95 g, 56.9 mmol, 2.60 mmol) and Meldrum´s acid (3.55 g, 24.6 mmol, 1.12 equiv) were added at room temperature and the resulting mixture was stirred for another 1 h before it was diluted with diethyl ether (200 mL). The organic phase was washed with 1M aqueous solution of hydrochloric acid (3 × 50 mL) and brine (50 mL). Then, the organic phase was dried over anhydrous magnesium sulfate, the dried solution was filtered and the filtrate was concentrated. Obtained residue was dissolved in methanol (60 mL) and it was heated to reflux and stirred for 16 h. Then, the mixture was allowed to cool down to room temperature, the solvent was evaporated and the obtained residue was passed through a short pad of silica gel (dichloromethane–ethyl acetate 10:1) to provide keto-ester **2** as a yellowish oil.

The intermediate **2** (3.51 g, 12.3 mmol, 1 equiv) was dissolved in toluene (11 mL) and 3-aminopyrazole (1.02 g, 12.3 mmol, 1.00 mmol) was added in one portion at room temperature. The resulting mixture was heated to reflux and stirred for 24 h. Then, the mixture was allowed to cool down to room temperature, the solvent was evaporated and the obtained residue was purified by flash-column

[1] SCH900776 was prepared by procedures described in patent literature (Paruch, K.; Guzi, T. J.; Dwyer, M. P.; Alvarez, C. S. WO 2007/041712 A1, 2007.) with minor modifications.

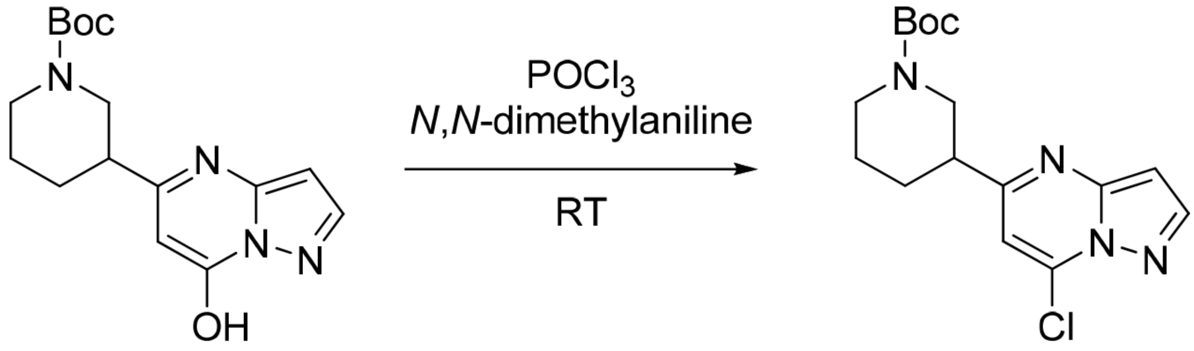

3 **4**

chromatography (gradient elution with dichloromethane–methanol 20:1 → 12:1) to provide product **3** (3.11 g, 45% from **1**) as a yellowish solid. ^{1}H NMR (300 MHz, $CDCl_3$) δ: 11.85 (bs, 1H), 7.83 (d, *J* = 1.5 Hz, 1H), 6.14 (d, *J* = 1.5 Hz, 1H), 5.75 (s, 1H), 4.07 (m, 1H), 3.81 (m, 1H), 3.33 (m, 1H), 2.84–3.15 (m, 2H), 2.16 (m, 1H), 1.86 (m, 1H), 1.65 (m, 1H), 1.53 (m, 1H), 1.44 (s, 9H).

Compound 4

N,*N*-Dimethylaniline (3.84 mL, 30.3 mmol, 3.1 equiv) was added to a stirred solution of compound **3** (3.11 g, 9.77 mmol, 1 equiv) in phosphorus oxychloride (6.90 mL) at room temperature. The resulting mixture was stirred at this temperature for 4 days. Then, excess of phosphorous oxychloride was evaporated. The obtained residue was carefully poured into saturated aqueous solution of sodium bicarbonate (200 mL) and the aqueous phase was extracted with dichloromethane (3 × 80 mL). The combined organic extracts were dried over anhydrous magnesium sulfate, the dried solution was filtered, and the filtrate was concentrated. The residue was purified by flash-column chromatography (dichloromethane–ethyl acetate 8:1) to provide product **4** (2.30 g, 70%) as a yellow wax. ^{1}H NMR (300 MHz, $CDCl_3$) δ: 8.18 (d, *J* = 2.0 Hz, 1H), 6.91 (s, 1H), 6.71 (d, *J* = 2.0 Hz, 1H), 4.28 (m, 1H), 4.10 (m, 1H), 3.08 (m, 1H), 2.78–2.96 (m, 2H), 2.00–2.17 (m, 1H), 1.74–1.91 (m, 2H), 1.59–1.68 (m, 1H), 1.47 (s, 9H).

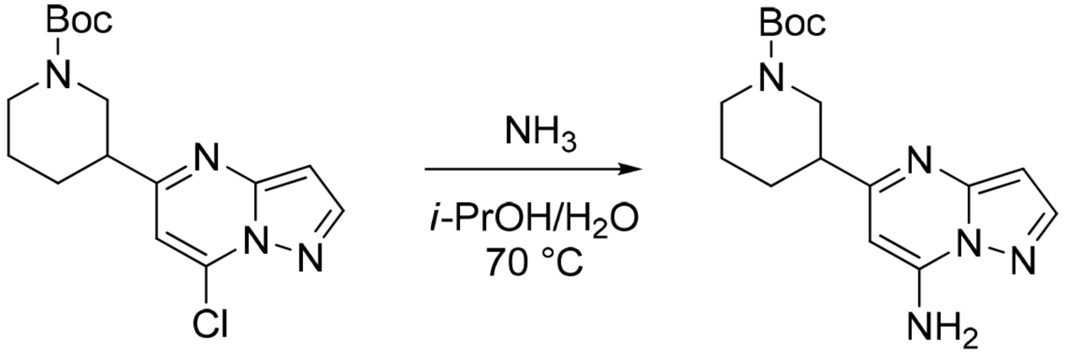

4 **5**

Compound 5

Compound **4** (2.30 g, 6.83 mmol, 1 equiv) was mixed with 2M solution of ammonia in propan-2-ol (14 mL) and 29% aqueous solution of ammonia (1.4 mL) at room temperature in a pressure tube. The resulting mixture was stirred at 70°C for 30 h. Then, the mixture was allowed to cool down to room temperature, the solvents were evaporated and the obtained residue was purified by flash-column chromatography (dichloromethane–methanol 10:1) to provide product **5** (1.97 g, 90%) as a white solid. ^{1}H NMR (300 MHz, $CDCl_3$) δ: 8.01 (d, *J* = 1.9 Hz, 1H), 6.45 (d, *J* = 1.9 Hz, 1H), 6.01 (s, 1H), 5.57 (s, 2H), 4.26 (m, 1H), 4.11 (m, 1H), 3.03 (m, 1H), 2.68–2.86 (m, 2H), 2.06 (m, 1H), 1.69–1.90 (m, 2H), 1.53–1.64 (m, 1H), 1.47 (s, 9H).

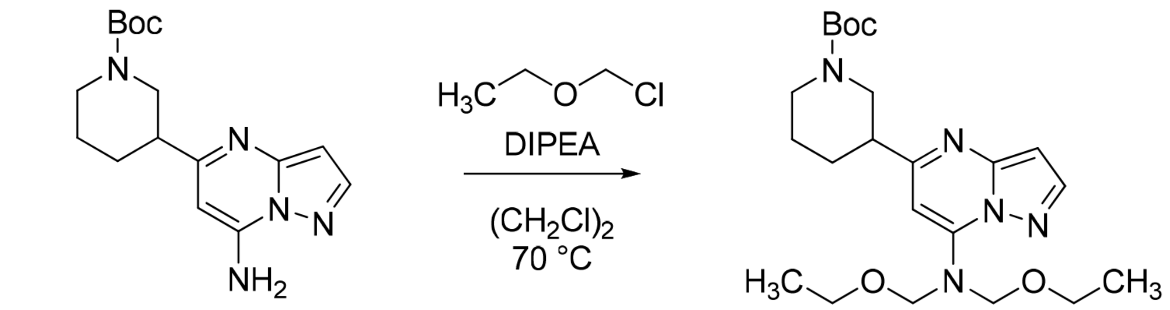

5 **6**

Compound 6

N,*N*-diisopropylethylamine (7.58 mL, 43.5 mmol, 7.0 equiv) and chloromethyl(ethyl) ether (2.02 mL, 21.7 mmol, 3.5 equiv) were added to a stirred solution of compound **5** (1.97 g, 6.21 mmol, 1 equiv) in 1,2-dichloroethane (20 mL) at room temperature. The resulting mixture was stirred at 70°C for 4 h. Then, the mixture was allowed to cool down to room temperature and it was diluted with dichloromethane (50 mL) and saturated aqueous solution of sodium bicarbonate (50 mL). The phases were separated and the aqueous phase was extracted with dichloromethane (2 × 50 mL). The combined organic extracts were dried over anhydrous magnesium sulfate, the dried solution was filtered and the filtrate was concentrated. The residue was purified by flash-column chromatography (dichloromethane–ethyl acetate 2:1) to provide product **6** as a yellow solid (2.29 g, 85%). ^{1}H NMR (300 MHz, $CDCl_3$) δ: 7.99 (d, *J* = 2.0 Hz, 1H), 6.49 (d, *J* = 2.0 Hz, 1H), 6.39 (s, 1H), 5.24 (m, 4H), 4.26 (m, 1H), 4.10 (m, 1H), 3.57 (q, *J*= 7.0 Hz, 4H), 3.01 (m, 1H), 2.68–2.82 (m, 2H), 2.05 (m, 1H), 1.67–1.90 (m, 2H), 1.53–1.63 (m, 1H), 1.44 (s, 9H), 1.17 (t, *J* = 7.0 Hz, 6H)

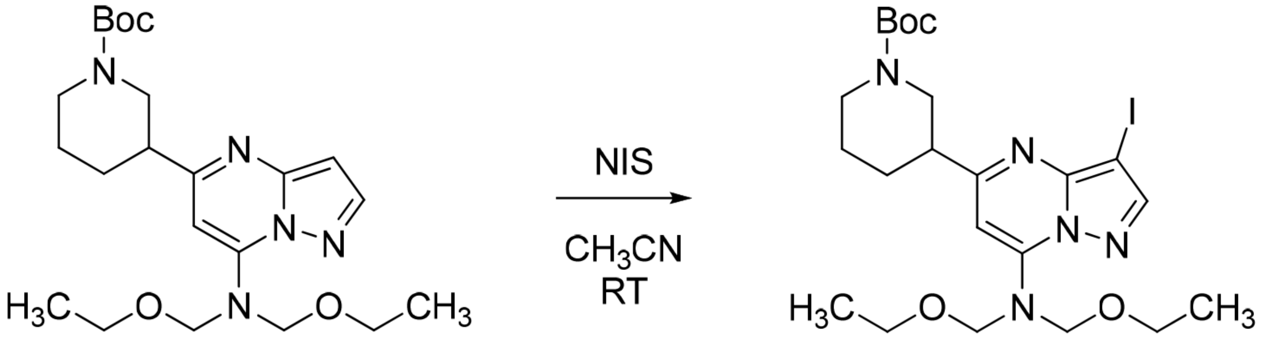

6 **7**

Compound 7

N-Iodosuccinimide (778 mg, 3.46 mmol, 1.0 equiv) was added to a stirred solution of compound **6** (1.50 g, 3.46 mmol, 1 equiv) in acetonitrile (18 mL) at room temperature. The resulting mixture was stirred at this temperature for 90 min in the dark. Then, the reaction mixture was concentrated and the residue was purified by flash-column chromatography (dichloromethane–ethyl acetate 15:1) to provide product **7** as a yellow wax (1.74 g, 90%). ^{1}H NMR (300 MHz, $CDCl_3$) δ: 8.01 (s, 1H), 6.46 (s, 1H), 5.23 (m, 4H), 4.32 (m, 1H), 4.13 (m, 1H), 3.58 (q, *J*= 7.0 Hz, 4H), 3.07 (m, 1H), 2.74–2.92 (m, 2H), 2.09 (m, 1H), 1.73–1.90 (m, 2H), 1.54–1.65 (m, 1H), 1.47 (s, 9H), 1.19 (t, *J* = 7 Hz, 6H).

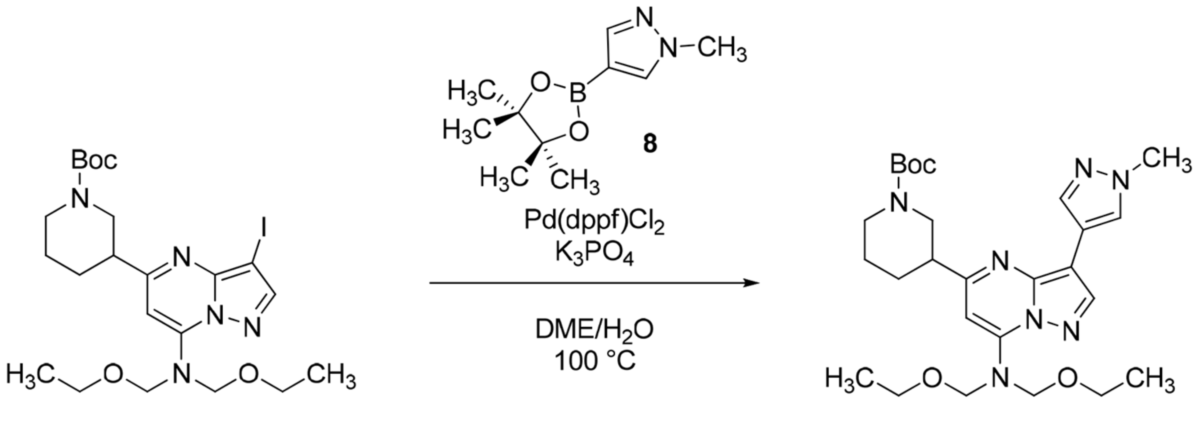

7 9

Compound 9

A reaction flask thoroughly purged with argon was charged with compound **7** (1.60 g, 2.86 mmol, 1 equiv), boronate **8** (1.19 g, 5.72 mmol, 2.0 equiv), [1,1′-bis(diphenylphosphino)ferrocene] dichloropalladium(II) catalyst (209 mg, 0.286 mmol, 0.1 equiv) and tripotassium phosphate (2.43 g, 11.4 mmol, 4.0 equiv). The mixture of reagents was suspended in 1,2-dimethoxyethane (30 mL) and water (6 mL) at room temperature. The resulting mixture was heated to 100 °C and stirred at this temperature for 4 h. Then, the mixture was allowed to cool down to room temperature, and water (60 mL) with ethyl acetate (50 mL) were added. The phases were separated and the aqueous phase was extracted with ethyl acetate (2 × 50 mL). The combined organic extracts were dried over anhydrous magnesium sulfate, the dried solution was filtered, and the filtrate was concentrated. The residue was purified by flash-column chromatography (dichloromethane–ethyl acetate 3:1) to provide product **9** as a yellow solid (734 mg, 50%). ^{1}H NMR (300 MHz, $CDCl_3$) δ: 8.16 (s, 1H), 7.98 (s, 1H), 7.96 (s, 1H), 6.42 (s, 1H), 5.27 (m, 4H), 4.37 (m, 1H), 4.17 (m, 1H), 3.99 (s, 3H), 3.60 (q, J = 7.0 Hz, 4H), 3.10 (m, 1H), 2.72–2.87 (m, 2H), 2.13 (m, 1H), 1.74–1.91 (m, 2H), 1.53–1.67 (m, 1H), 1.48 (s, 9H), 1.20 (t, J = 7.0 Hz, 6H).

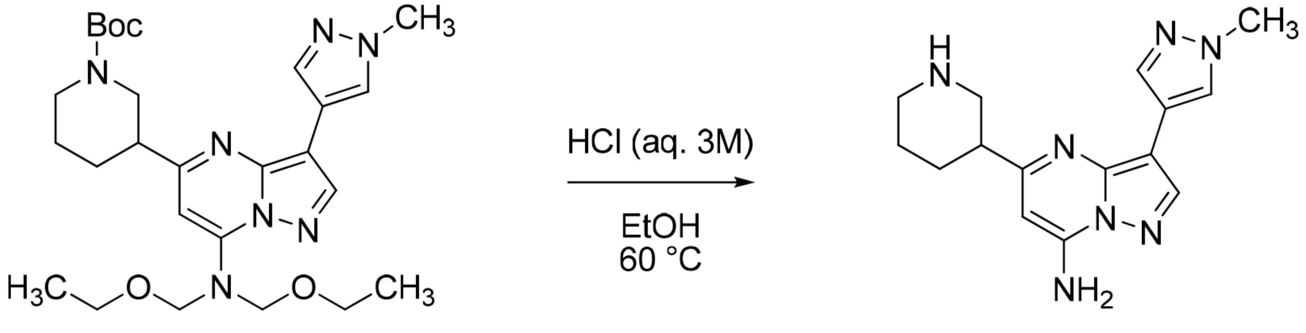

9 10

Compound 10

An aqueous solution of hydrochloric acid (3M, 15 mL) was added to a stirred solution of compounds **9** (734 mg, 1.43 mmol, 1 equiv) in ethanol (15 mL) at room temperature. The resulting solution was heated to 60 °C and stirred at this temperature for 2 h. Then, the solution was allowed to cool down to room temperature, the solvents were evaporated and the obtained residue was dissolved in dichloromethane–methanol mixture (5:1, 15 mL). To the mixture was added solid sodium carbonate (2.0 g) and the suspension was stirred at room temperature for 30 min. The mixture was then passed through a short pad of silica gel (dichloromethane–7M solution of ammonia in methanol 6:1) to provide product **10** as a white solid (365 mg, 86%). ^{1}H NMR (300 MHz, DMSO-d_6) δ: 8.27 (s, 1H), 8.06 (s, 1H), 7.91 (s, 1H), 7.52 (s, 2H), 5.98 (s, 1H), 3.98 (s, 3H), 3.11 (m, 1H), 2.92 (m, 1H), 2.64–2.84 (m, 2H), 2.52 (m, 1H), 2.0 (m, 1H), 1.44–1.79 (m, 3H).

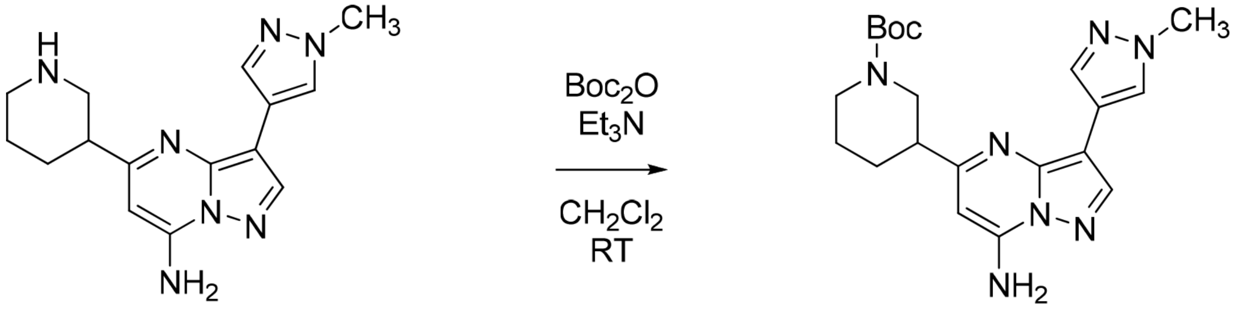

10 **11**

Compound 11

Di-*tert*-butyl dicarbonate (264 mg, 1.21 mmol, 1.2 equiv) was added to a stirred solution of compound **10** (300 mg, 1.01 mmol, 1 equiv) in dichloromethane (6 mL) and triethylamine (1.2 mL) at room temperature. After stirring at this temperature for 18 h, the mixture was diluted with dichloromethane (30 mL) and saturated aqueous solution of sodium bicarbonate (40 mL). The phases were separated and the aqueous phase was extracted with dichloromethane (2 × 30 mL). The combined organic extracts were dried over anhydrous magnesium sulfate, the dried solution was filtered, and the filtrate was concentrated. The residue was purified by flash-column chromatography (dichloromethane–methanol 20:1) to provide product **11** as a yellow solid (321 mg, 80%). ^{1}H NMR (300 MHz, $CDCl_3$) δ: 8.15 (s, 1H), 7.98 (s, 1H), 7.94 (s, 1H), 6.01 (s, 1H), 5.51 (s, 2H), 4.33 (m, 1H), 4.10 (m, 1H), 3.98 (s, 3H), 3.10 (m, 1H), 2.70–2.90 (m, 2H), 2.14 (m, 1H), 1.77–1.95 (m, 2H), 1.53–1.64 (m, 1H), 1.48 (s, 9H).

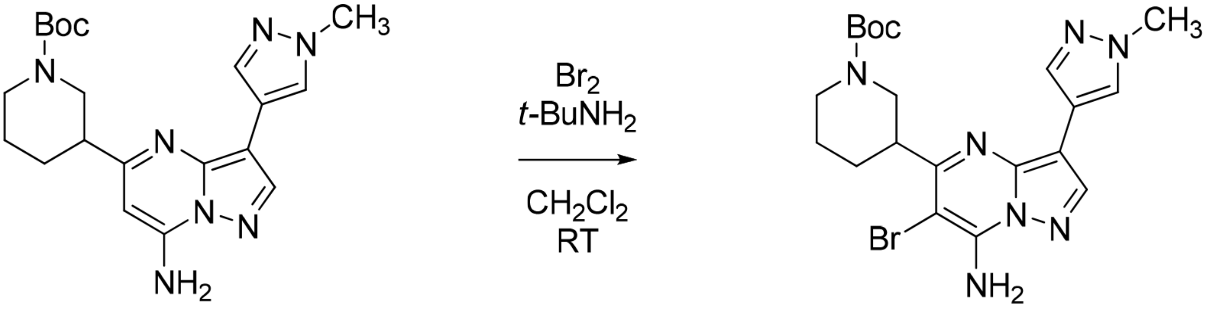

11 **12**

Compound 12

A solution of bromine (25 µL, 0.478 mmol, 1 equiv) in dichloromethane was added to a stirred solution of compound **11** (190 mg, 0.478 mmol, 1 equiv) in *tert*-butylamine (4 mL) and dichloromethane (2 mL) at room temperature. The resulting mixture was stirred at this temperature for 20 h. Then, the reaction mixture was concentrated in a vacuum and the residue was purified by flash-column chromatography (dichloromethane–ethyl acetate 1:1) to provide product **12** as a white solid (192 mg, 84%). ^{1}H NMR (300 MHz, $CDCl_3$) δ: 8.12 (s, 1H), 7.95 (s, 1H), 7.92 (s, 1H), 6.01 (s, 2H), 4.32 (m, 1H), 4.20 (m, 1H), 3.99 (s, 3H), 3.25 (m, 1H), 3.13 (m, 1H), 2.80 (m, 1H), 2.10 (m, 1H), 1.83 (m, 2H), 1.62 (m, 1H), 1.48 (s, 9H).

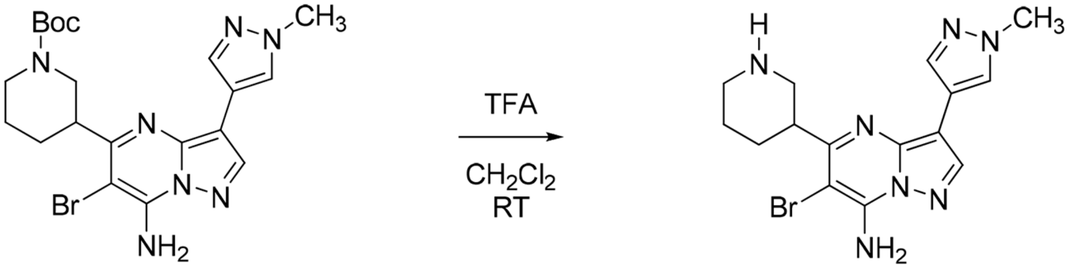

12 **(±)-SCH900776**

Racemic SCH900776

Trifluoroacetic acid (2 mL) was added to a stirred solution of compound **12** (181 mg, 0.380 mmol, 1 equiv) in dichloromethane (2 mL) at room temperature. The resulting mixture was stirred at this temperature for 1 h. Then, the solvent was evaporated and the obtained residue was dissolved in methanol (4 mL). To the mixture was added solid sodium carbonate (500 mg) and the suspension was stirred at room temperature for 30 min. The mixture was then passed through a short pad of silica gel (dichloromethane–7M solution of ammonia in methanol 15:1) to provide racemic SCH900776 as a white solid (135 mg, 94 %). ^{1}H NMR (300 MHz, DMSO-d_6) δ: 8.32 (s, 1H), 8.06 (s, 1H), 7.94 (s, 1H), 3.89 (s, 3H), 3.05–3.22 (m, 2H), 2.96 (m, 1H), 2.77 (m, 1H), 2.54 (m, 1H), 1.96 (m, 1H), 1.66–1.85 (m, 2H), 1.52 (m, 1H). ^{13}C NMR (75 MHz, DMSO-d_6) δ: 160.16, 145.17, 142.27, 140.83, 135.71, 126.41, 112.94, 101.11, 83.22, 50.66, 46.01, 43.47, 38.53, 29.54, 26.14. IR (KBr), cm^{-1}: 3429, 3274, 2937, 1640, 1586, 1541, 1341, 1250, 1212. HRMS (APCI) calcd for $[C_{15}H_{18}N_7Br+H]^+$: 376.0880/378.0861, found: 376.0875/378.0856. Melting point = 214.5–215.7 °C.

The enantiomers were separated using Chiralcel® OJ™ column; flow: 20 mL/min; injection: 5 mL of racemate solution in EtOH (4 mg/mL); mobile phase: n-hexane/ethanol 80:20 + 0.5 % diethylamine. **SCH900776** is the faster eluting enantiomer (retention time: 10. 04 min).

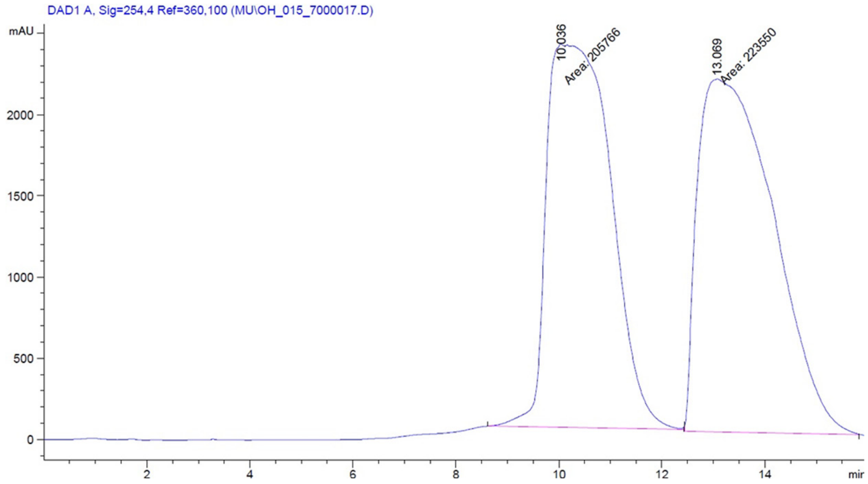

SUPPLEMENTARY METHOD S2: GATING OF LEUKEMIC CELLS IN THE FLOW CYTOMETRY ANALYSIS IN TCL1-MICE

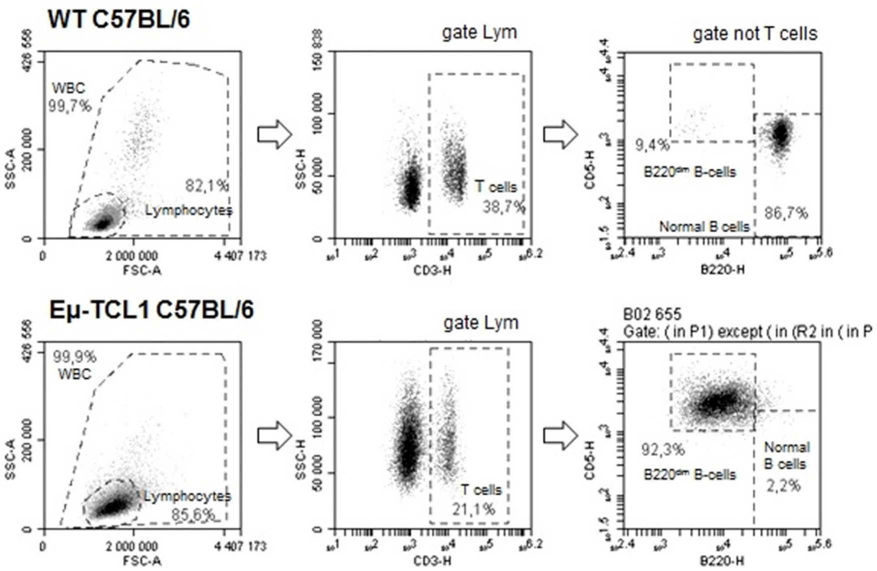

To demonstrate the difference between wt C57BL/6 animals and adult Eμ-TCL1 C57BL/6 mice with progressed B-cell leukemia and to explain the process of leukemic B-cell quantification, we describe respective flow cytometry analysis. Peripheral blood samples were analyzed using BD Accuri C6 flow cytometer as follows: Red blood cells (RBC) were lysed by NH_4Cl lysis buffer and the remaining white blood cells (WBC) were washed and stained according to the manufacturer´s instructions using CD5, B220 (CD45R) and CD3 primary antibodies conjugated with fluorochromes. Stained cells were washed by PBS and resuspended in the final volume of 100 µL PBS. Absolute quantification of the cell counts was achieved by calibration of the measurement by flow-cytometry absolute count standard (Bangs Laboratories). Singlets were determined and then lymphocytes were gated according to their morphology (images i, iv; low FSC and SSC). T-cells were excluded using CD3 staining (ii, v) and then leukemic ($B220^{dim}$) and normal B-cells were discriminated using B220 and CD5 staining (iii, vi). Mice with progressed leukemia typically lack the normal B-cell population, while the $B220^{dim}CD5^{+}$ cells are dominant.

REFERENCES

1. Wallace PK, Muirhead KA. Cell tracking 2007: a proliferation of probes and applications. Immunol Invest. 2007; 36:527-61.
2. Dietrich S, Krämer OH, Hahn E, Schäfer C, Giese T, Hess M, Tretter T, Rieger M, Hüllein J, Zenz T, Ho AD, Dreger P, Luft T. Leflunomide induces apoptosis in fludarabine-resistant and clinically refractory CLL cells. Clin Cancer Res. 2012; 18:417-31.
3. Willimott S, Baou M, Naresh K, Wagner SD. CD154 induces a switch in pro-survival Bcl-2 family members in chronic lymphocytic leukaemia. Br J Haematol. 2007; 138:721-32.
4. Williamson CT, Muzik H, Turhan AG, Zamò A, O'Connor MJ, Bebb DG, Lees-Miller SP. ATM deficiency sensitizes mantle cell lymphoma cells to poly(ADP-ribose) polymerase-1 inhibitors. Mol Cancer Ther. 2010; 9:347-57.

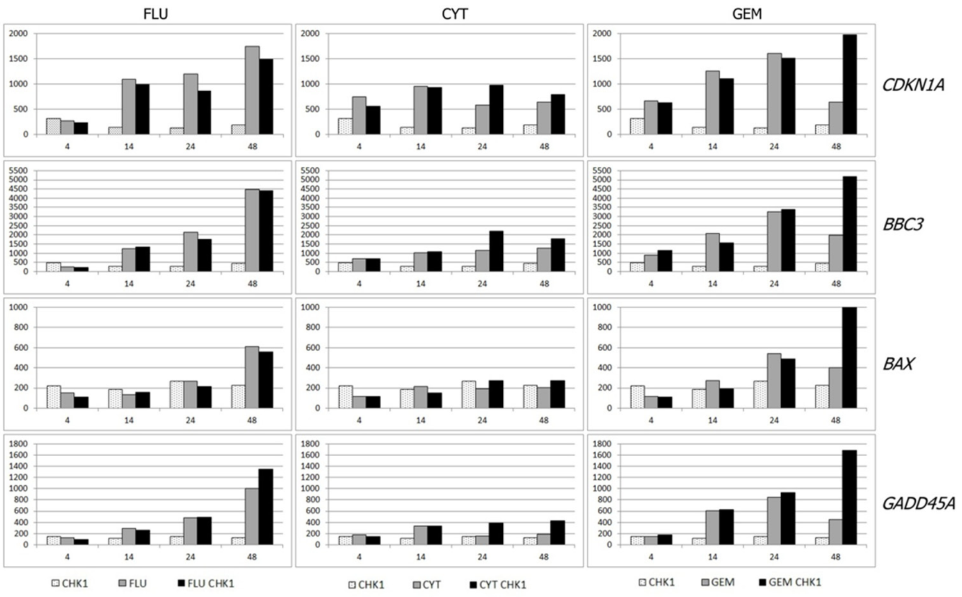

Supplementary Figure S1A: Real time PCR analysis of the p53 pathway induction in NALM-6 cell line. Boxes from left to right show treatments with FLU, CYT, and GEM at individual time points (h). The columns represent SCH900776 alone (white), indicated NA (gray), and NA with the Chk1 inhibitor (black). The expression of genes analyzed after treatments represents a change compared to the corresponding untreated control (set as 100% and not shown in the graphs). The Chk1 inhibitor on its own induced only negligible *CDKN1A* gene induction: 4 h 311%; 14 h 135%; 24 h 127%; 48 h 182%. All tested p53 target genes, *CDKN1A*, *BBC3*, *BAX*, and *GADD45A*, exhibited substantially stronger induction in most of the treatments involving NAs, with or without the Chk1 inhibitor.

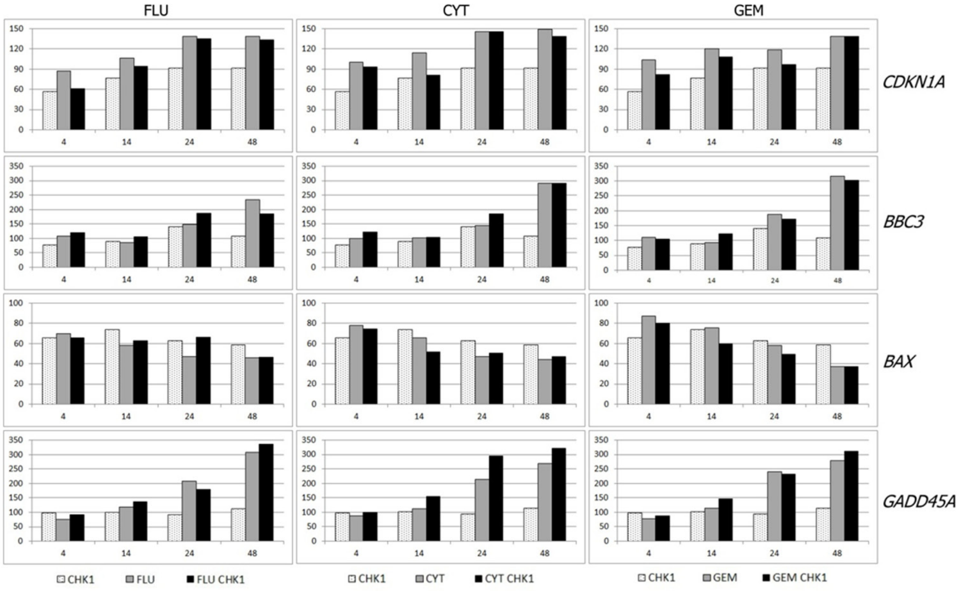

Supplementary Figure S1B: Real time PCR analysis of the p53 pathway induction in MEC-1 cell line. Boxes from left to right show treatments with FLU, CYT, and GEM at individual time points (h). The columns represent SCH900776 alone (white), indicated NA (gray), and NA with the Chk1 inhibitor (black). The expression of analyzed genes after treatments represents a change compared to corresponding untreated control (set as 100%). The analysis recorded only mild induction of *BBC3* and *GADD45A* genes after treatments, and no stimulation at all in the case of *CDKN1A* and *BAX*. The results confirm that p53-mutated cell line lacks induction of some of the critical cell cycle and apoptosis regulatory genes in studied treatments.

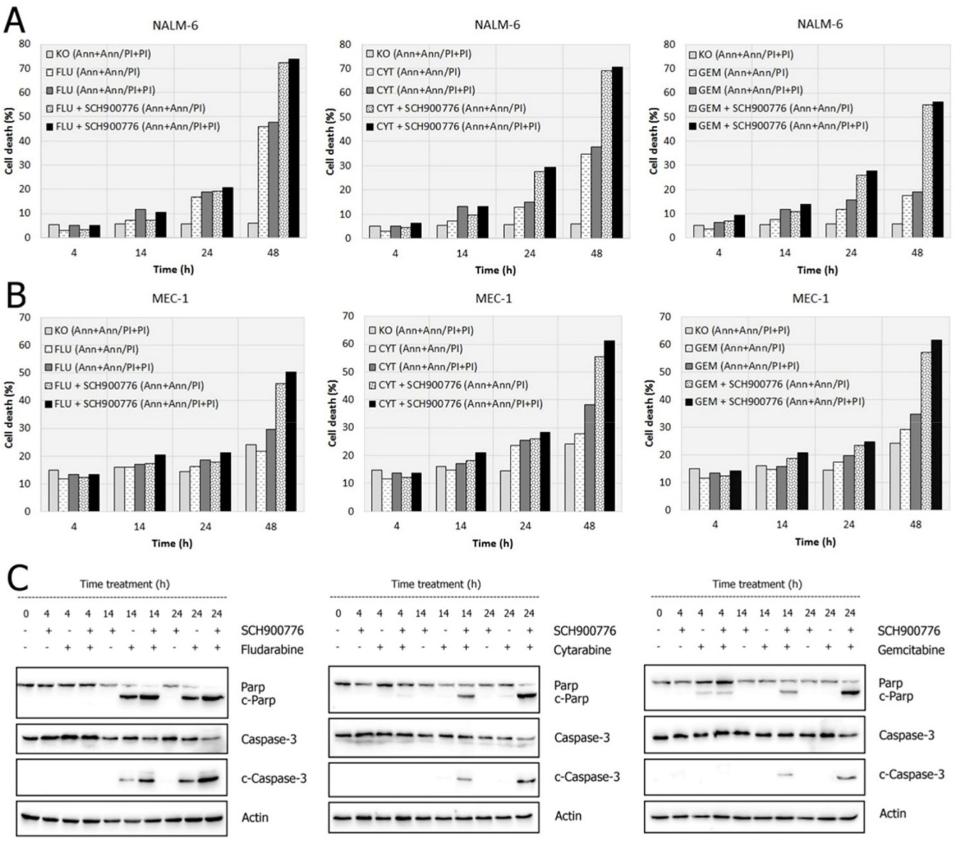

Supplementary Figure S2: Analysis of apoptosis. The graphs for NALM-6 **(A)** and MEC-1 **(B)** cell lines summarize the results of flow-cytometric analysis based on Annexin-V/PI staining, and show proportions of apoptotic (Ann+Ann/PI) and total dead (Ann+Ann/PI+PI) cells. In p53-wt NALM-6 cell line, all three NAs elicited concentration-dependent increase of apoptotic cell proportion with further augmentation in Chk1-inhibited co-treatments. In p53-mutated MEC-1 cell line, the effects of NAs were poor and massive apoptosis was present only when the Chk1 protein was inhibited. In NALM-6 cell line, the increase of apoptosis through SCH900776 was confirmed by WB **(C)** showing cleaved PARP (c-PARP) and cleaved caspase-3 (c-caspase-3) proteins. The interval 48 h was not analyzed because of the strongly damaged cells in co-treatments with SCH900776.

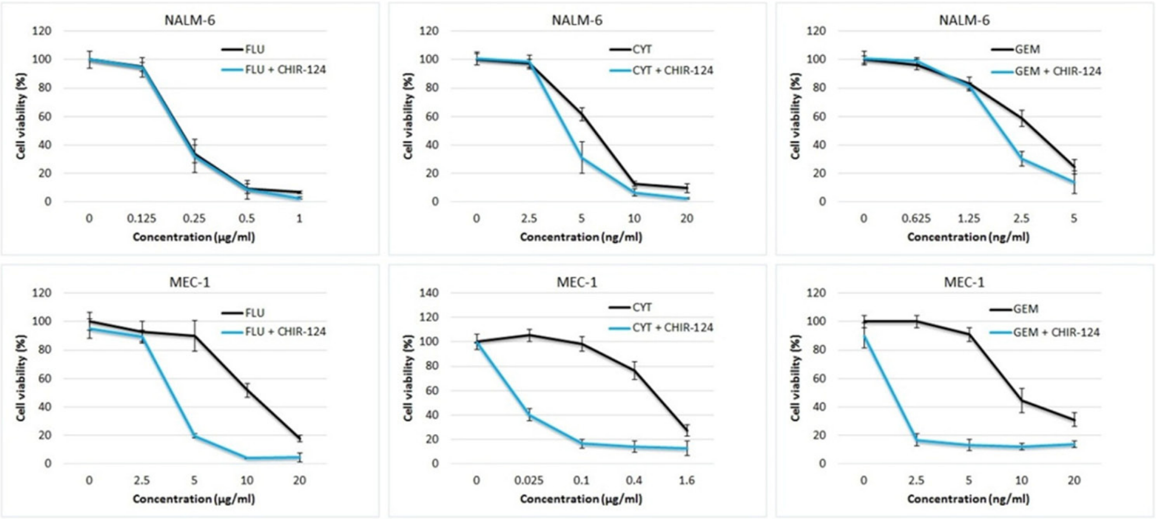

Supplementary Figure S3: Impact of Chk1 inhibitor CHIR-124 on the viability of NALM-6 and MEC-1 cell lines treated with fludarabine. Graphs show viability curves obtained in the metabolic WST-1 assay. Experimental design was the same as in the parallel experiment analyzing SCH900776, including the overall time of treatment (72 h) and NAs concentrations (stated in the graphs). CHIR-124 was used at the final concentration 50 nM. In p53-mutated MEC-1 cells, this structurally unrelated Chk1 inhibitor exhinited very similar synergistic effect with FLU as SCH900776.

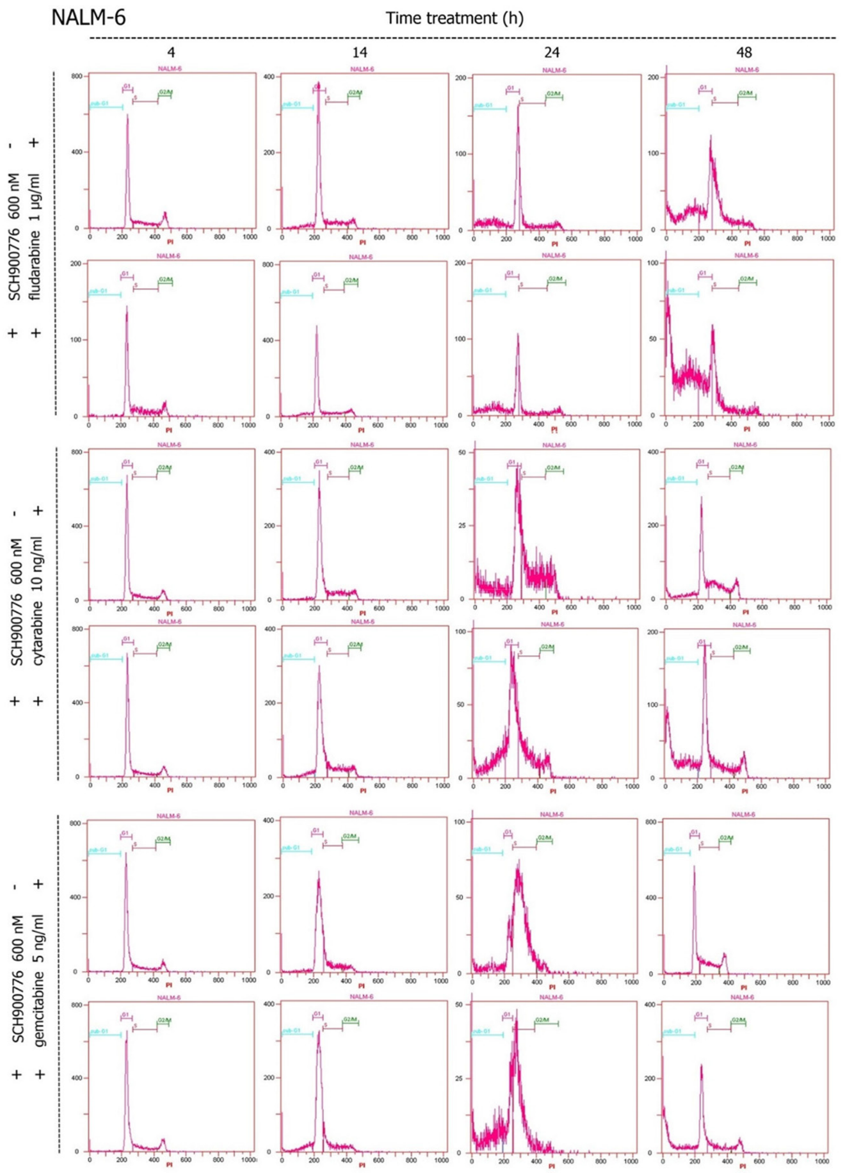

Supplementary Figure S4: Flow cytometry analysis of the cell cycle in NALM-6 cell line. Time intervals from the left are 4 h, 14 h, 24 h, and 48 h. Cell cycle and apoptotic changes are described in the text and in Supplementary Table S1.

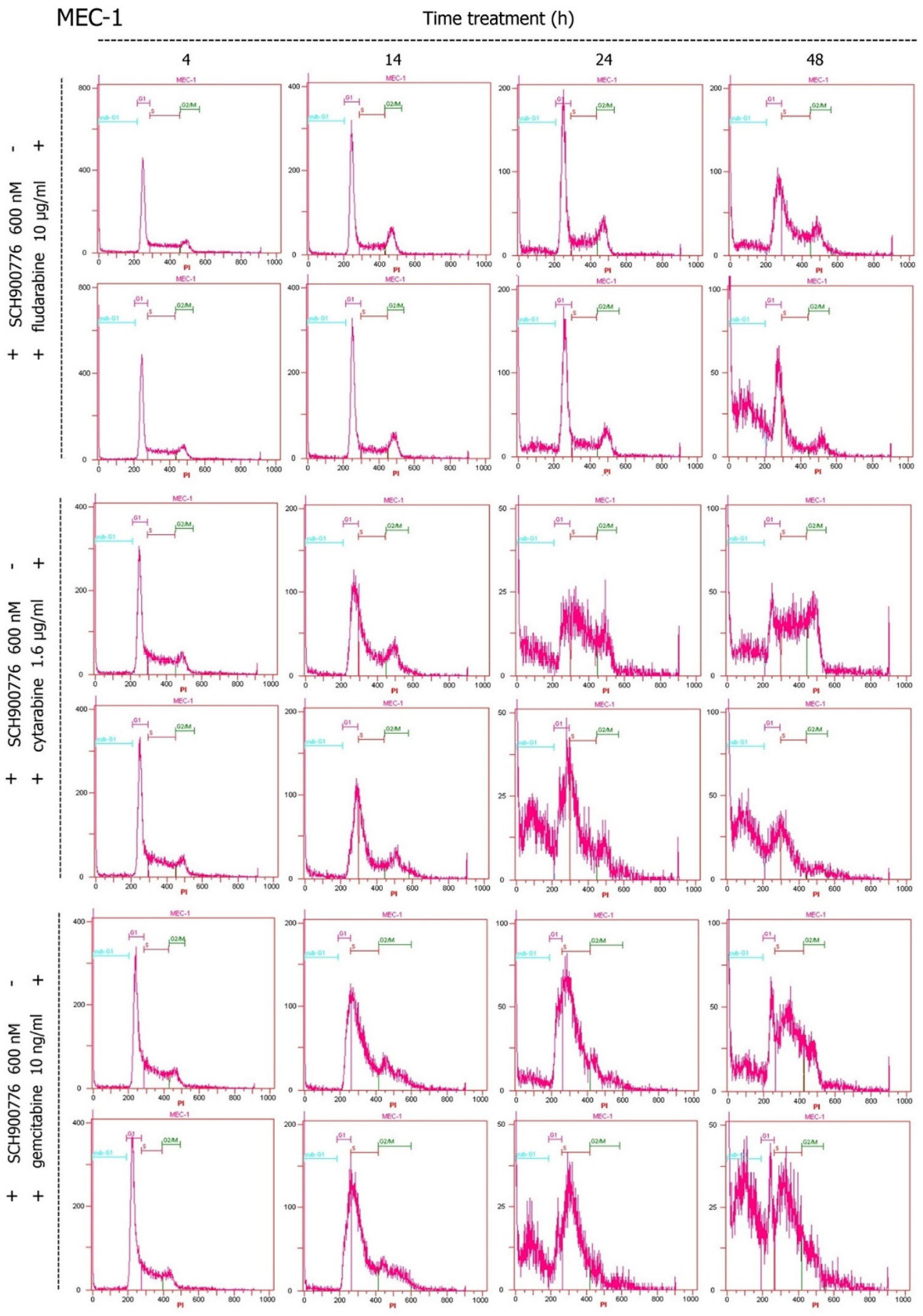

Supplementary Figure S5: Flow cytometry analysis of the cell cycle in MEC-1 cell line. Time intervals from the left are 4 h, 14 h, 24 h, and 48 h. Cell cycle and apoptotic changes are described in the text and in Supplementary Table S2.

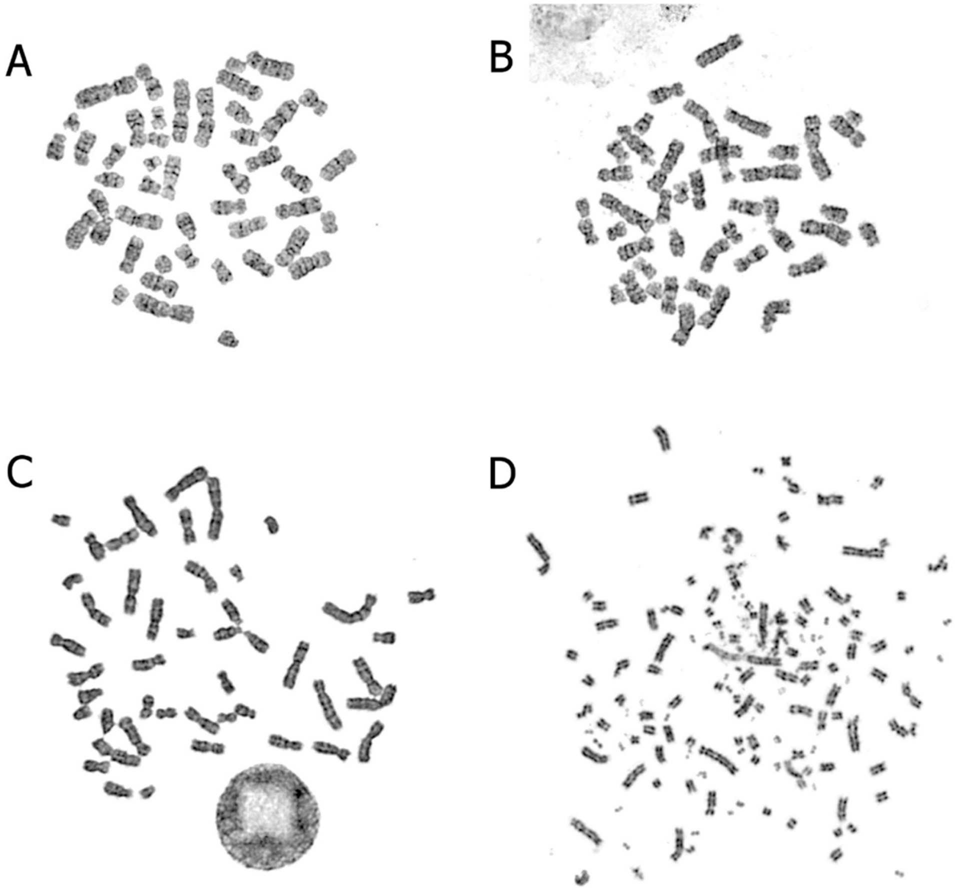

Supplementary Figure S6: Cytogenetic analyses of NALM-6 and MEC-1 cell lines treated with SCH900776 and fludarabine. In NALM-6 cell line, we did not record any difference between the untreated **(A)** and treated **(B)** cells. By contrast, MEC-1 cell line cultured in the presence of SCH900776 and FLU **(D)** showed an extensive chromosome fragmentation (pulverization), not observed in control untreated cells **(C)**. In both cell lines, treatments resulted in a substantially reduced mitotic index counted from 1000 cells: in MEC-1, control 15.8%, FLU 0.8%, and FLU with Chk1 inhibitor 0.6%; in NALM-6, control 21%, FLU 5.4%, and FLU with Chk1 inhibitor 7.7%.

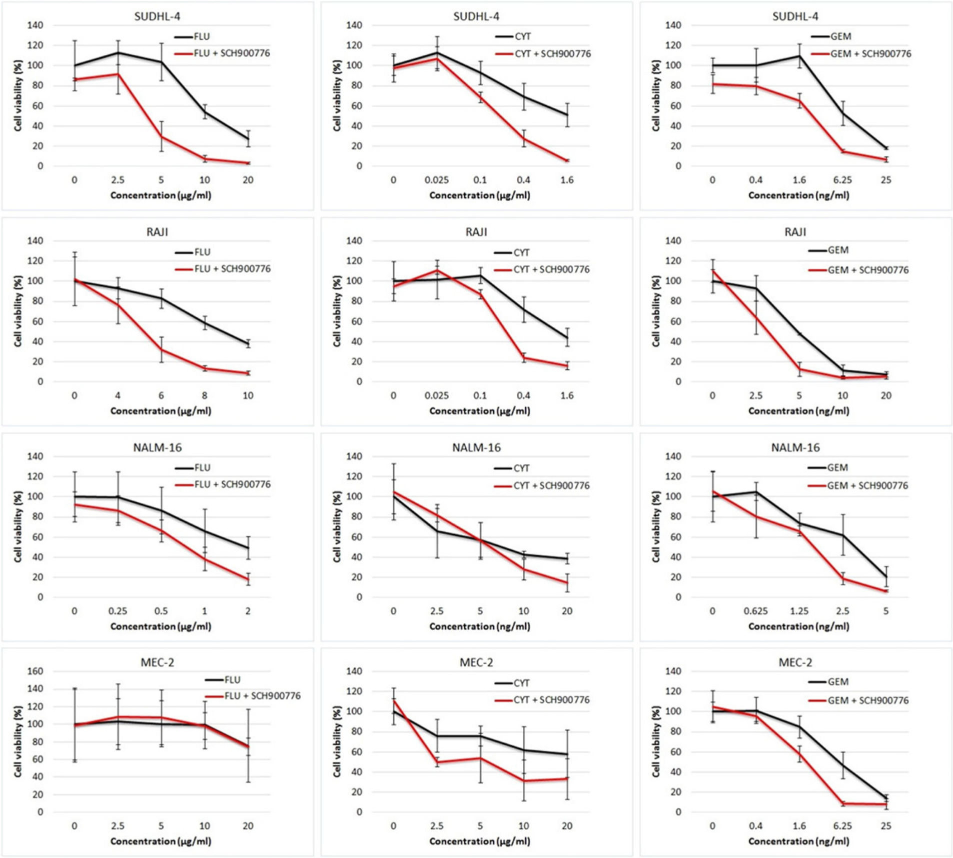

Supplementary Figure S7A: Impact of SCH900776 administration on the viability of SUDHL-4, RAJI, NALM-16, and MEC-2 cell lines treated with NAs. Statistical evaluation of the effects is available in Table 2.

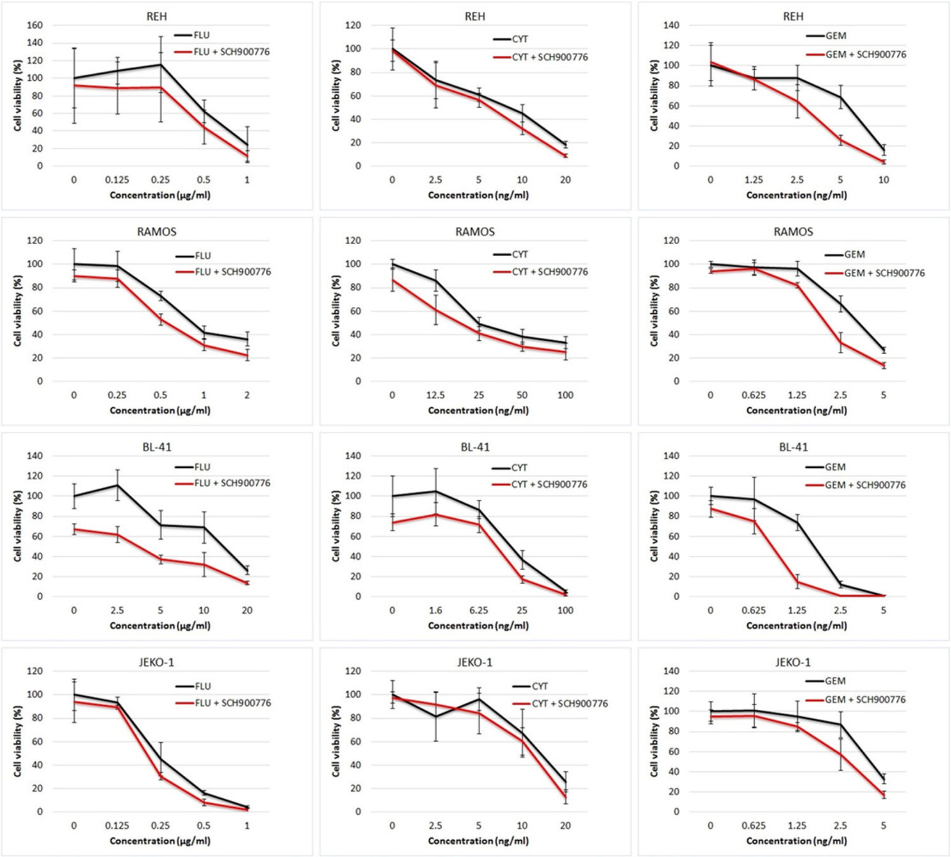

Supplementary Figure S7B: Impact of SCH900776 administration on the viability of REH, RAMOS, BL-41, and JEKO-1 cell lines treated with NAs. Statistical evaluation of the effects is available in Table 2.

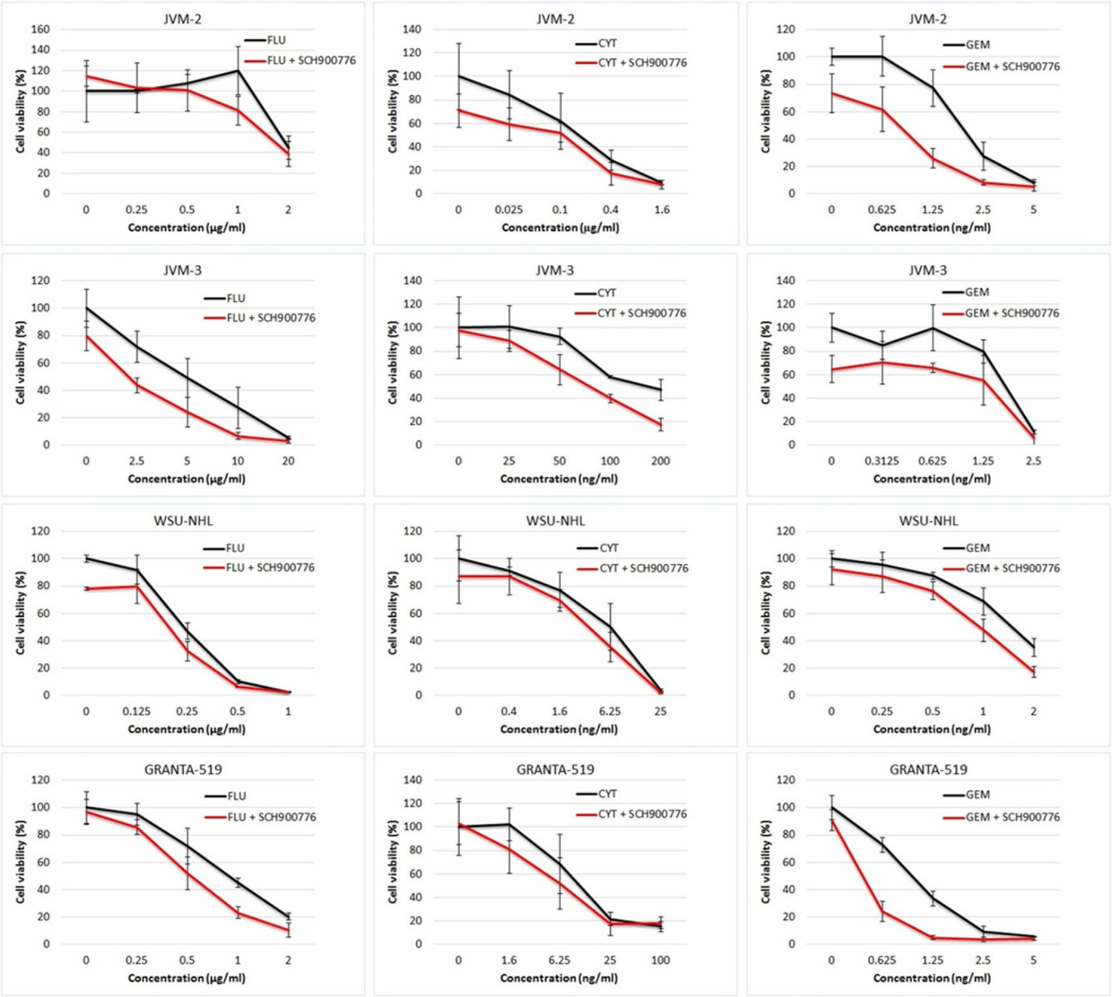

Supplementary Figure S7C: Impact of SCH900776 administration on viability of JVM-2, JVM-3, WSU-NHL, and GRANTA-519 cell lines treated with NAs. Statistical evaluation of the effects is available in Table 2.

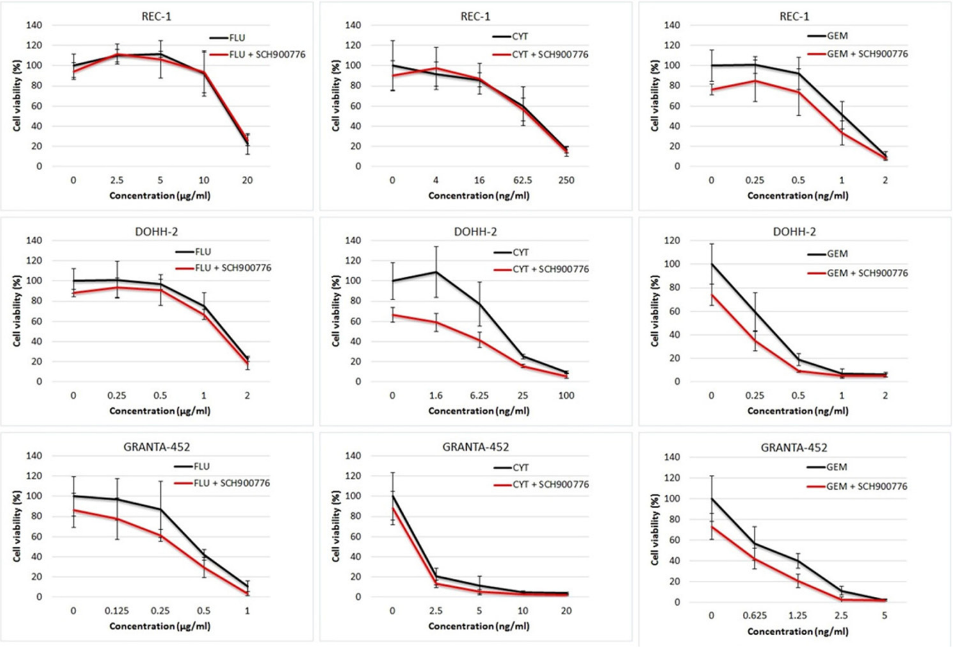

Supplementary Figure S7D: Impact of SCH900776 administration on the viability of REC-1, DOHH-2, and GRANTA-452 cell lines treated with NAs. Statistical evaluation of the effects is available in Table 2.

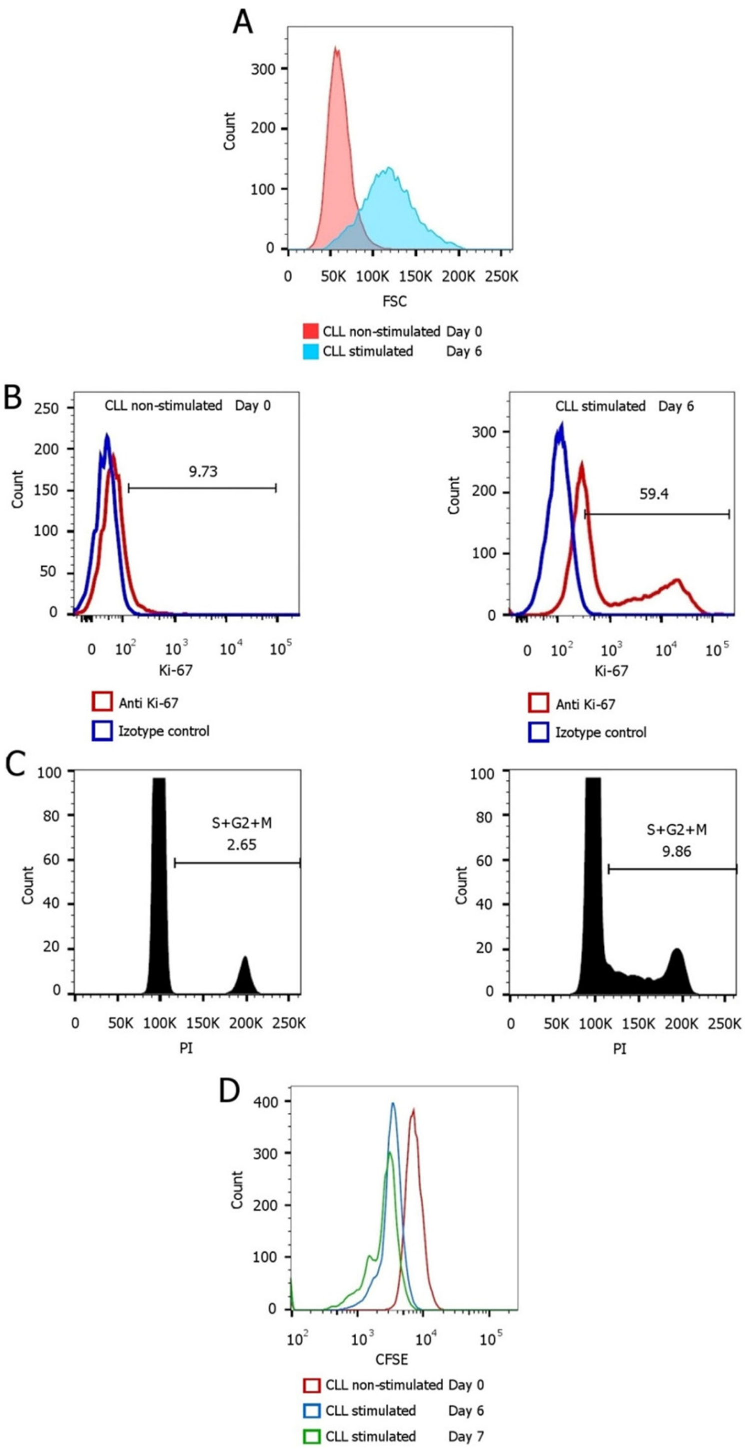

Supplementary Figure S8: Phenotype and molecular changes associated with pro-proliferative stimulation of CLL cells. After the six-day co-culture with CD32-transfected murine L-cells in the presence of anti-CD40 antibody and IL-4, CLL cells significantly enlarged their size as evidenced by forward scatter in flow-cytometry (FSC) **(A)** increased expression of Ki-67 protein **(B)** and manifested proliferative fraction in the cell cycle analysis **(C)** and in the CFSE dilution staining **(D)** In the last analysis, shift of the color to the left reflects CFSE dilution to daughter cells as consequence of cell division; see minor leftmost peaks for days 6 and 7. The major peaks measured on days 6 and 7 are shifted relative to the peak on day 0 due to the initial drop of CFSE intensity as previously described [1]. The additional time point "day 7 after stimulation" supplements data measured on day 6 and reveals further stimulation of CLL cells. All analyses in this figure were done using sample CLL9 from Table 3 and Figure 5. Parallel analyses (A-D) were done also on sample CLL1 with very similar results.

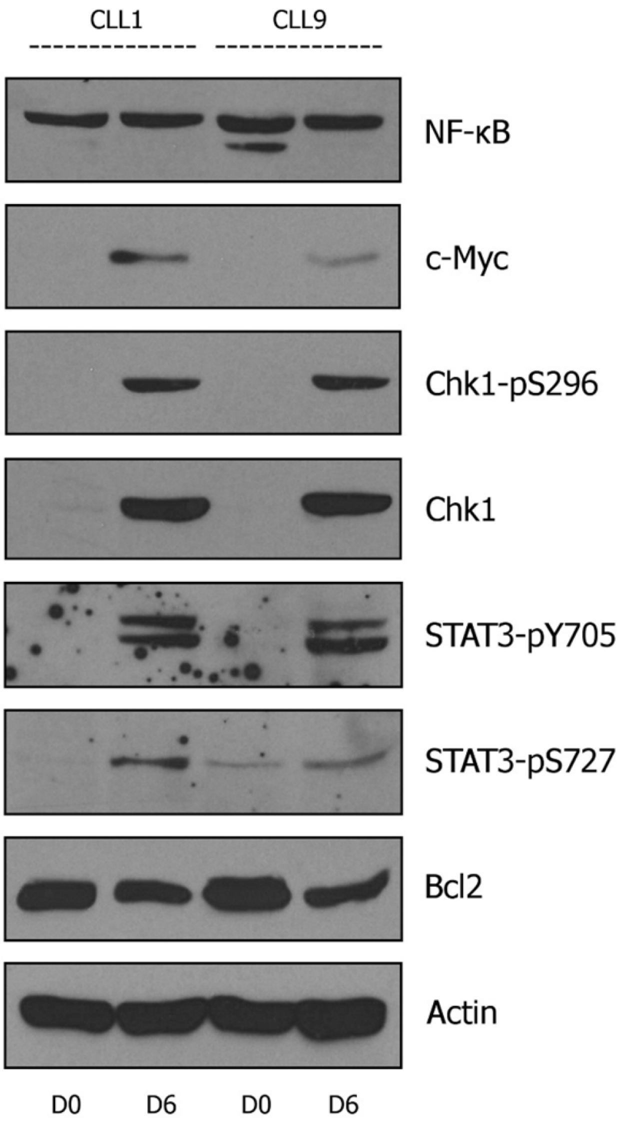

Supplementary Figure S9: Western blot analysis in non-stimulated and stimulated CLL cells. The level of selected proteins was assessed in the paired samples consisting of non-stimulated CLL cells on day 0 (D0) and CLL cells treated with pro-proliferative stimuli harvested on day 6 (D6). 50 µg of protein lysates were run on 10% SDS-PAGE gels. The samples designation corresponds to Table 3 and Figure 5. The blot demonstrates induction of total and activated Chk1 protein (Chk1 and Chk1-pS296), c-Myc and activated STAT3 (signal transducer and activator of transcription 3) protein (STAT3-pY705 and STAT3-pS727). All these changes were in line with the activated and pro-proliferative phenotype of CLL cells [2]. A decrease of Bcl-2 level after the stimulation also confirms previous observation by Willimott et al [3].

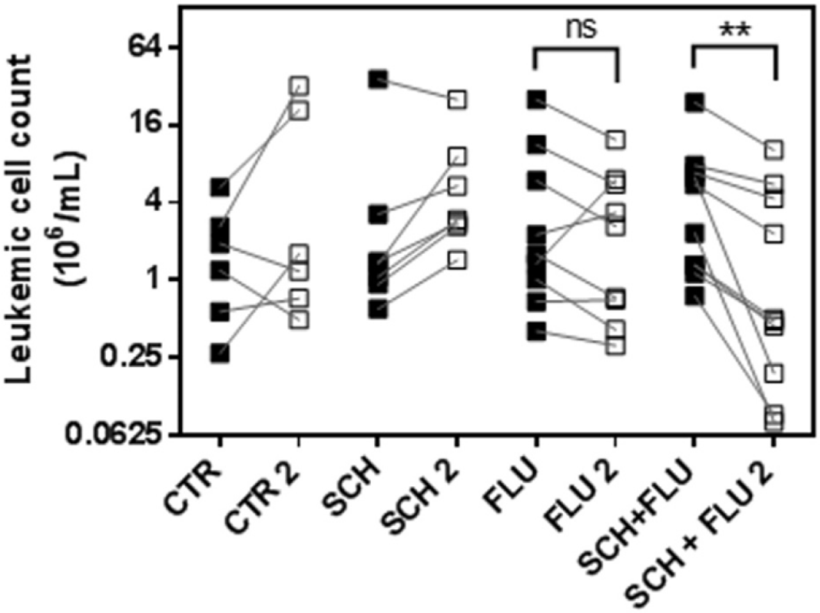

Supplementary Figure S10: Changes in leukemic cell numbers in the mouse Eμ-TCL1 model of CLL. Absolute leukemic cell counts detected in the peripheral blood during the samplings before and after (labelled "2") the 5-day treatment in the individual animals. Only the combined administration of SCH900776 with FLU caused significant decrease in leukemic cell counts (Wilcoxon signed rank test, P=0.0020). CTR: untreated control mice; SCH: Chk1 inhibitor SCH900776; FLU: fludarabine; SCH+FLU: inhibitor SCH900776 and fludarabine.

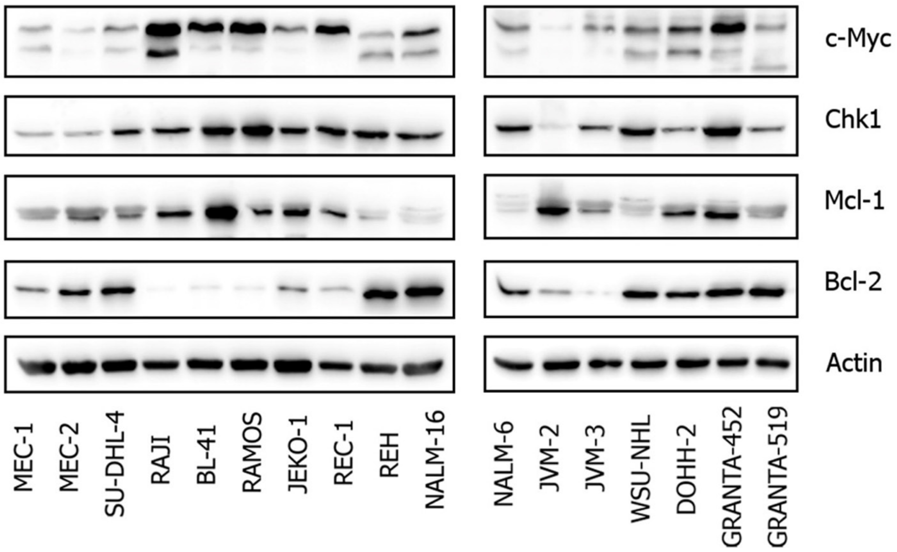

Supplementary Figure S11: Baseline level of selected proteins in the studied B-lymphoid cell lines. Western blot was done using 50 µg of protein lysates and 10% SDS-PAGE gels.

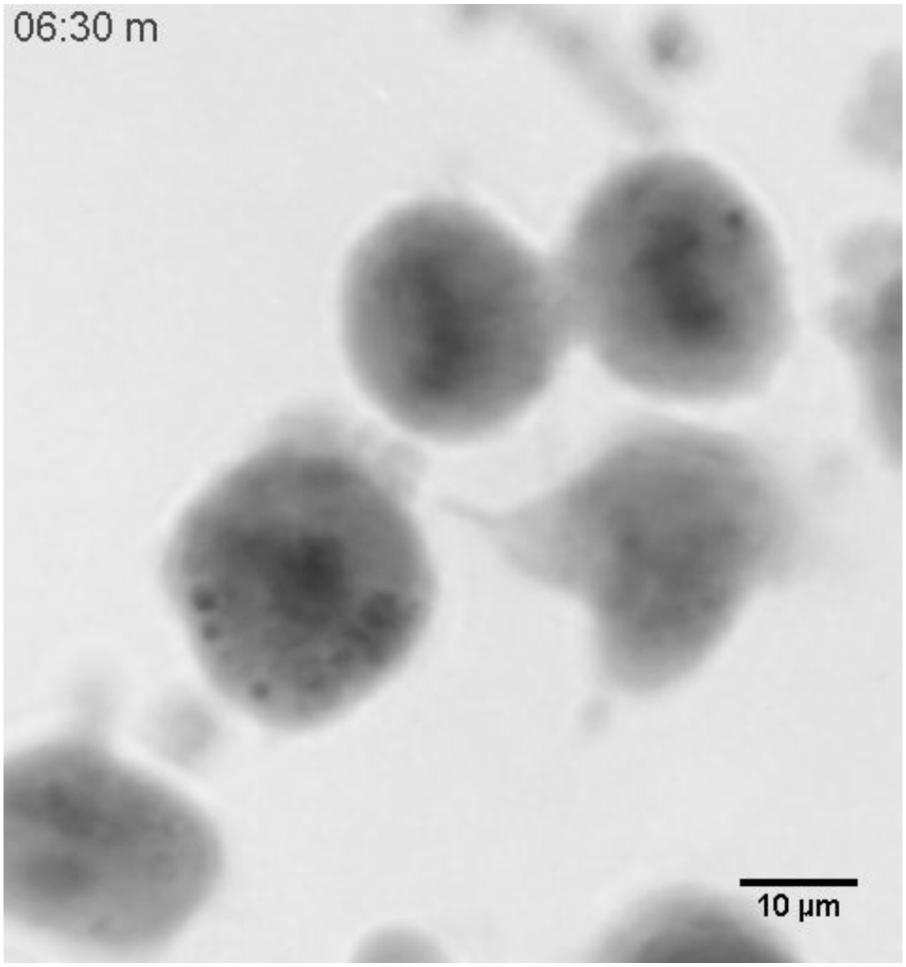

Supplementary video 1: Standard mitosis in untreated MEC-1 cells.

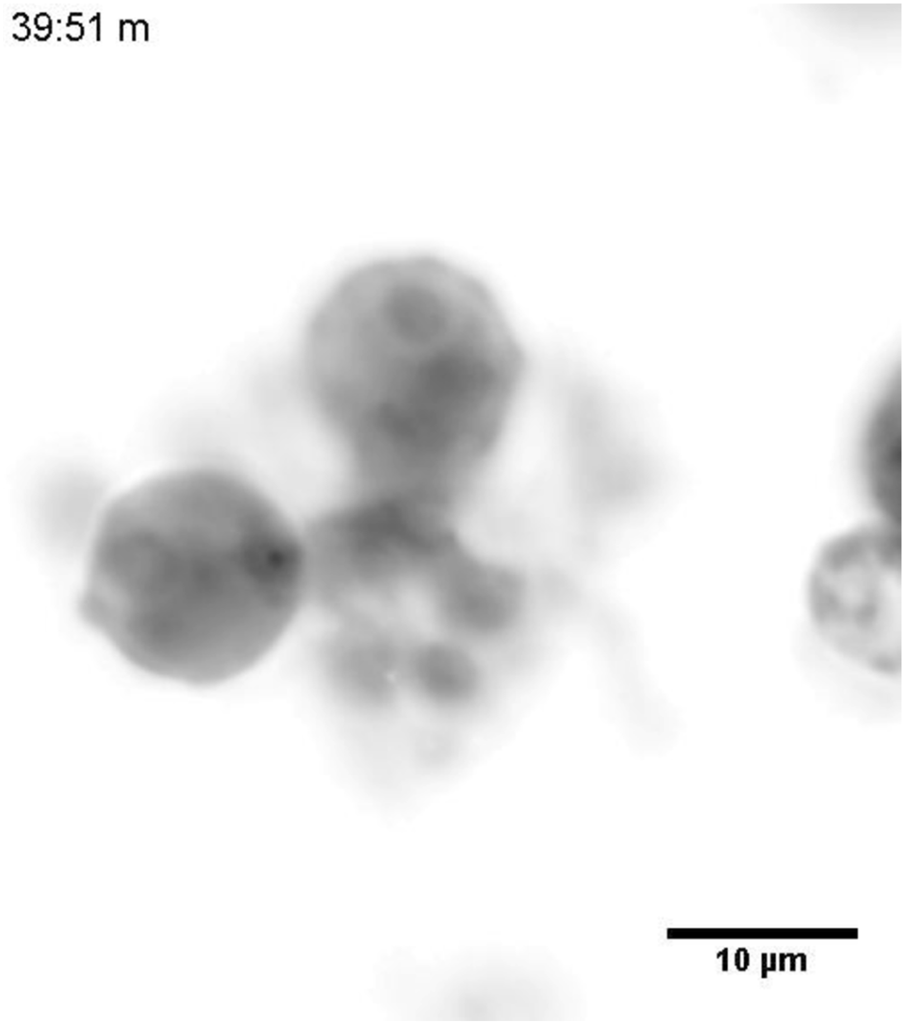

Supplementary video 2: Aberrant mitosis in treated MEC-1 cells.

Supplementary Table S1: The cell cycle analysis in NALM-6 cell line

NALM-6	Time (h)	Sub-G1 phase (%)	G1 phase (%)	S phase (%)	G2/M phase (%)
Control	4	1	56	24	18
	14	2	55	25	18
	24	2	57	23	18
	48	2	54	28	15
Fludarabine	4	2	59	24	15
	14	9	67	17	8
	24	26	45	21	8
	48	34	26	34	6
Fludarabine + SCH900776	4	3	59	24	13
	14	12	60	15	11
	24	30	41	20	8
	48	60	19	18	3
Cytarabine	4	2	70	17	11
	14	5	68	19	8
	24	19	36	31	12
	48	14	41	32	14
Cytarabine + SCH900776	4	2	70	16	12
	14	9	63	21	8
	24	24	44	24	7
	48	40	35	15	10
Gemcitabine	4	2	70	16	10
	14	5	67	20	8
	24	10	13	70	5
	48	5	48	31	16
Gemcitabine + SCH900776	4	2	71	16	11
	14	11	67	16	6
	24	26	21	49	4
	48	37	38	16	9

Table shows proportions of the cells at individual time points with respect to cell cycle phases and corresponding treatments.

Supplementary Table S2: The cell cycle analysis in MEC-1 cell line

MEC-1	Time (h)	Sub-G1 phase (%)	G1 phase (%)	S phase (%)	G2/M phase (%)
**Control**	4	5	51	16	23
	14	5	46	25	19
	24	9	45	22	19
	48	11	43	17	21
**Fludarabine**	4	7	48	25	15
	14	11	49	18	19
	24	19	44	18	16
	48	22	26	33	15
**Fludarabine + SCH900776**	4	13	45	25	12
	14	11	52	17	17
	24	25	42	15	15
	48	55	21	13	6
**Cytarabine**	4	9	45	28	14
	14	13	33	33	17
	24	44	12	27	12
	48	30	16	28	19
**Cytarabine + SCH900776**	4	8	47	28	13
	14	18	25	32	17
	24	47	19	21	8
	48	59	14	18	5
**Gemcitabine**	4	7	49	27	11
	14	8	18	50	21
	24	22	17	47	13
	48	29	13	39	14
**Gemcitabine + SCH900776**	4	7	54	23	12
	14	7	22	48	21
	24	47	10	35	7
	48	48	12	28	9

Table shows proportions of the cells at individual time points with respect to cell cycle phases and corresponding treatments.

Supplementary Table S3: Genetics of the B-lymphoid cell lines including *TP53* mutational status.

CLL/PL: chronic lymphocytic leukemia in prolymphocytoid transformation; DLBCL: diffuse large B-cell lymphoma; BL: Burkitt lymphoma; MCL: mantle cell lymphoma; ALL: acute lymphoblastic leukemia; B-PLL: B-cell prolymphocytic leukemia; FL: follicular lymphoma. The cell line GRANTA-519 harbors previously described ATM mutation p.R2832C together with heterozygous deletion 11q [4].

See Supplementary File 1

Supplementary Table S4: (A) Kinases with the final activity <30% in the *in vitro* testing of 1 µM SCH900776

Kinase	Final activity	Activity in DDR	Uniprot code
CHK1(h)	0	yes	O14757
CaMKIIδ(h)	3	no	Q13557
CaMKIIγ(h)	4	no	Q95MF5
Pim-3(h)	7	no	Q86V86
Pim-1(h)	14	no	P11309
Pim-2(h)	14	no	Q9P1W9
TLK2(h)	15	yes	Q86UE8
Rsk3(h)	17	no	Q15349
Haspin(h)	19	no	Q8TF76
SIK(h)	24	no	P57059
Rsk2(h)	26	no	P51812
CaMKIIβ(h)	27	no	P08413
Rsk4(h)	28	yes	Q9UK32

Supplementary Table S4: (B) Kinases with the final activity ≥ 30% in the *in vitro* testing of 1 µM SCH900776

See Supplementary File 1
