# Supplementary material for: Chk1 inhibition significantly potentiates activity of nucleoside analogs in TP53-mutated B-lymphoid cells
Source: Oncotarget. 2016 Aug 19;7(38):62091–106. doi: 10.18632/oncotarget.11388 (PMC5308713; doi:10.18632/oncotarget.11388)
Supplement: Supplementary file 2 [file oncotarget-07-62091-s002.docx]

**Supplementary Table S3. Genetics of the B-lymphoid cell lines including *TP53* mutational status.**

CLL/PL: chronic lymphocytic leukemia in prolymphocytoid transformation; DLBCL: diffuse large B-cell lymphoma; BL: Burkitt lymphoma; MCL: mantle cell lymphoma; ALL: acute lymphoblastic leukemia; B-PLL: B-cell prolymphocytic leukemia; FL: follicular lymphoma. The cell line GRANTA-519 harbors previously described ATM mutation p.R2832C together with heterozygous deletion 11q [4].

| Cell lines | Malignancy | *TP53* mutation | 17p- | *ATM* status | Other important  genomic aberrations | Cytogenetics | Doubling time (h) |
| --- | --- | --- | --- | --- | --- | --- | --- |
| MEC-1 | CLL/PL | c.949dupC; p.Q317fs | yes | WT |  | 46(44-47)˂2n˃XY | 40 |
| MEC-2 | CLL/PL | c.949dupC; p.Q317fs | yes | WT |  | 46(44-47)˂2n˃XY | 40 |
| SU-DHL-4 | DLBCL | c.817C˃T; p.R273C | yes | WT | t(14;18) (*IGH / BCL2*) | 50(47-51)˂2n˃XY/XXY | 40 |
| RAJI | BL | c.638G˃A; p.R213Q  c.700T˃C; p.Y234H | yes | WT | t(8;14) (*MYC / IGH*) | 89(80-91)˂4n˃XXYY | 24-36 |
| BL-41 | BL | c.743G˃A; p.R248Q | yes | WT | t(8;14) (*MYC / IGH*) | 48(42-49)˂2n˃XY | 30 |
| RAMOS | BL | c.760_761AT>GA; p.I254D | yes | WT | t(8;14) (*MYC / IGH*) | 45(44-46)˂2n˃X | 48 |
| JEKO-1 | MCL | c.173delC; p.P58* | yes | WT | t(11;14) (*CCND1 / IGH*) | 70-78˂3n˃XXXX | 50 |
| REC-1 | MCL | c.734 G˃A; p.G245D  c. 949 C˃T; p.Q317* | yes | WT | t(11;14) (*CCND1 / IGH*) | 41-47˂2n˃XY/XXY | 20-40 |
| REH | ALL | c.541C˃T; p.R181C | no | WT | t(12;21) (*TEL / AML1*) | 46(44-47)˂2n˃X | 50-70 |
| NALM-16 | ALL | c.868_869insTC; p.R290fs | n.a. | WT |  | 52(49-53)˂2n˃XX | 36 |
| NALM-6 | ALL | WT | no | WT | t(5;12) (*ETV6 / PDGFRB*) | 46(43-47)˂2n˃XY | 36 |
| JVM-2 | B-PLL | WT | no | WT | t(11;14) (*CCND1 / IGH*) | 46(42-46)˂2n˃XX | 50-70 |
| JVM-3 | B-PLL | WT | no | WT |  | 87-90˂4n˃XXYY | 30 |
| WSU-NHL | DLBCL | WT | no | WT | t(14;18) (*IGH / BCL2*) |  | 45 |
| DOHH-2 | FL | WT | no | WT | t(8;14;18) (*MYC / IGH / BCL2*) | 47(43-48)˂2n˃XY | 40 |
| GRANTA-452 | ALL | WT | no | WT | t(9;14;18) (*PAX5 / IGH / BCL2*) t(8;22) (*MYC / IGL*) | 49(46-50) ˂2n˃XXY | 48 |
| GRANTA-519 | MCL | WT | yes | MUT | t(11;14) (*CCND1 / IGH*) | 44(39-44)˂2n˃XX | 49 |

**Supplementary Table S4.**

(B) Kinases with the final activity ≥30% in the *in vitro* testing of 1 µM SCH900776.

| Kinase | Final activity |
| --- | --- |
| CaMKIδ(h) | 30 |
| LRRK2(h) | 34 |
| MELK(h) | 39 |
| CLK4(h) | 45 |
| PASK(h) | 45 |
| LOK(h) | 46 |
| Rsk1(h) | 47 |
| AMPKα1(h) | 51 |
| ARK5(h) | 52 |
| CK2(h) | 53 |
| Mer(h) | 54 |
| NLK(h) | 57 |
| CaMKI(h) | 60 |
| Ret(h) | 60 |
| TrkA(h) | 61 |
| PDK1(h) | 62 |
| Txk(h) | 62 |
| CLK2(h) | 63 |
| MEK1(h) | 63 |
| Pyk2(h) | 63 |
| CK2α2(h) | 64 |
| CLK1(h) | 64 |
| CDK5/p35(h) | 65 |
| CHK2(h) | 65 |
| Yes(h) | 65 |
| ACK1(h) | 66 |
| Lck(h) | 66 |
| AMPKα2(h) | 67 |
| RIPK2(h) | 67 |
| TSSK1(h) | 67 |
| CDK5/p25(h) | 68 |
| Flt1(h) | 68 |
| PRK2(h) | 69 |
| CDK2/cyclinA(h) | 70 |
| Flt4(h) | 70 |
| Bmx(h) | 71 |
| BrSK2(h) | 71 |
| Fms(h) | 72 |
| MARK1(h) | 72 |
| MuSK(h) | 72 |
| MST3(h) | 74 |
| FGFR1(h) | 75 |
| PDGFRβ(h) | 75 |
| GCK(h) | 76 |
| KDR(h) | 76 |
| CDK3/cyclinE(h) | 77 |
| CDK6/cyclinD3(h) | 77 |
| IRAK4(h) | 77 |
| Lyn(h) | 77 |
| ROCK-II(h) | 77 |
| Aurora-C(h) | 78 |
| BrSK1(h) | 78 |
| CK1γ1(h) | 78 |
| CK1δ(h) | 78 |
| CSK(h) | 78 |
| MLK1(h) | 78 |
| ULK2(h) | 78 |
| MAPK2(h) | 79 |
| ZIPK(h) | 79 |
| BRK(h) | 80 |
| CDK1/cyclinB(h) | 80 |
| Flt3(h) | 80 |
| IKKβ(h) | 80 |
| Ron(h) | 80 |
| CDK2/cyclinE(h) | 81 |
| cKit(h) | 81 |
| LKB1(h) | 81 |
| MST1(h) | 81 |
| NEK11(h) | 81 |
| Axl(h) | 82 |
| DCAMKL2(h) | 82 |
| Fgr(h) | 82 |
| IKKα(h) | 82 |
| PDGFRα(h) | 82 |
| PKD2(h) | 82 |
| SGK2(h) | 82 |
| WNK3(h) | 82 |
| DYRK2(h) | 83 |
| FGFR4(h) | 83 |
| Fyn(h) | 83 |
| MSK1(h) | 83 |
| PTK5(h) | 83 |
| TBK1(h) | 83 |
| c-RAF(h) | 84 |
| Hck(h) activated | 84 |
| p70S6K(h) | 84 |
| PKBγ(h) | 84 |
| LIMK1(h) | 85 |
| MSK2(h) | 85 |
| PAR-1Bα(h) | 85 |
| STK33(h) | 85 |
| ALK(h) | 86 |
| Blk(h) | 86 |
| CDK9/cyclin T1(h) | 86 |
| Itk(h) | 86 |
| MST2(h) | 86 |
| mTOR/FKBP12(h) | 86 |
| TSSK2(h) | 86 |
| WNK2(h) | 86 |
| FGFR2(h) | 87 |
| FGFR3(h) | 87 |
| IGF-1R(h), activated | 87 |
| IRR(h) | 87 |
| PKG1α(h) | 87 |
| ULK3(h) | 87 |
| cSRC(h) | 88 |
| FAK(h) | 88 |
| Hck(h) | 88 |
| MLCK(h) | 88 |
| Mnk2(h) | 88 |
| CDK7/cyclinH/MAT1(h) | 89 |
| Fes(h) | 89 |
| MINK(h) | 89 |
| PRAK(h) | 89 |
| ROCK-I(h) | 89 |
| TYK2(h) | 89 |
| Aurora-A(h) | 90 |
| CK1γ3(h) | 90 |
| DAPK1(h) | 90 |
| GSK3α(h) | 90 |
| JAK3(h) | 90 |
| Syk(h) | 90 |
| B-Raf(h) | 91 |
| HIPK2(h) | 91 |
| MRCKα(h) | 91 |
| Plk1(h) | 91 |
| Snk(h) | 91 |
| TAK1(h) | 91 |
| PIP5K1γ(h) | 91 |
| CaMKIV(h) | 92 |
| GRK7(h) | 92 |
| IR(h) | 92 |
| NEK3(h) | 92 |
| SGK(h) | 92 |
| JAK2(h) | 93 |
| PEK(h) | 93 |
| PKBα(h) | 93 |
| Tie2 (h) | 93 |
| DDR2(h) | 94 |
| EGFR(h) | 94 |
| IGF-1R(h) | 94 |
| JNK1α1(h) | 94 |
| Rse(h) | 94 |
| SGK3(h) | 94 |
| Wee1(h) | 94 |
| DMPK(h) | 95 |
| IKKε(h) | 95 |
| JNK3(h) | 95 |
| NEK2(h) | 95 |
| PKA(h) | 95 |
| PKG1β(h) | 95 |
| ASK1(h) | 96 |
| MRCKβ(h) | 96 |
| PAK1(h) | 96 |
| PhKγ2(h) | 96 |
| PIP4K2α(h) | 96 |
| CLK3(h) | 97 |
| DAPK2(h) | 97 |
| GRK6(h) | 97 |
| HIPK1(h) | 97 |
| HIPK3(h) | 97 |
| MAPK1(h) | 97 |
| SRPK2(h) | 97 |
| ZAP-70(h) | 97 |
| Abl(h) | 98 |
| DRAK1(h) | 98 |
| IRAK1(h) | 98 |
| JAK1(h) | 98 |
| MSSK1(h) | 98 |
| NEK7(h) | 98 |
| PKBβ(h) | 98 |
| VRK2(h) | 98 |
| GRK5(h) | 99 |
| GSK3β(h) | 99 |
| mTOR(h) | 99 |
| TGFBR1(h) | 99 |
| Arg(h) | 100 |
| Met(h) | 100 |
| PKCα(h) | 100 |
| PrKX(h) | 100 |
| TrkC(h) | 100 |
| eEF-2K(h) | 102 |
| NEK6(h) | 102 |
| PIP5K1α(h) | 102 |
| BTK(h) | 103 |
| TrkB(h) | 103 |
| SRPK1(h) | 104 |
| CK1γ2(h) | 105 |
| Plk3(h) | 105 |
| JNK2α2(h) | 106 |
| ALK4(h) | 108 |
| Ros(h) | 108 |
| Fer(h) | 109 |
| Aurora-B(h) | 139 |
